# Multi-Omics Analysis of *Phytolacca acinosa* Fruit Fermentation Broth Against Hepatocellular Carcinoma and Evaluation of Phytolaccagenin as a Candidate Bioactive Constituent

**DOI:** 10.3390/ijms27219620

**Published:** 2026-10-27

**Authors:** Yan Zou, Zhiqiang Li, Jiangang Cao, Yongwei Shu, Yusheng Zhou, Bikui Zhang

**Affiliations:** 1 The Second Xiangya Hospital of Central South University, Changsha 410011, China; 18907349998@163.com; 2 Hengyang Medical School, University of South China, Hengyang 421001, China

**Keywords:** *Phytolacca acinosa* fruit fermentation broth, hepatocellular carcinoma, multi-omics, UPLC-Q-TOF-MS/MS, active components

## Abstract

*Phytolacca acinosa* Roxb. is a traditional Chinese medicine. In traditional practice, its root has long been used for “promoting diuresis to reduce edema” and “detoxifying and resolving masses”, and certain constituents of the root have been reported to exhibit in vitro anti-tumor activities. However, the fruit of *Phytolacca acinosa* represents a newly explored medicinal resource, and the anti-hepatocellular carcinoma (HCC) activity of its fermentation broth (PAFFB) remains largely unexplored. This study evaluated the anti-HCC efficacy of PAFFB and its underlying mechanisms by integrating pharmacodynamics, multi-omics (transcriptomics, proteomics, and metabolomics), and UPLC-Q-TOF-MS/MS profiling. In a diethylnitrosamine (DEN)-induced HCC rat model, PAFFB (0.75, 1.5, and 3 g/kg/d for 4 weeks) dose-dependently improved body weight and survival, alleviated liver injury, reduced tumor burden, and restored liver function, while exhibiting less apparent toxicity than cyclophosphamide in the parameters assessed (body weight, survival, and hepatic function and histopathology). Joint pathway enrichment analysis revealed that PAFFB modulated DEN-induced pathological alterations through the coordinated regulation of cholesterol–bile acid metabolism, inflammatory signaling, and energy metabolism. Mechanistically, PAFFB restored TLR-4/MYD88 expression, suppressed p65 and p38 phosphorylation, and upregulated CYP7A1, PPARα, and FXR. Among the 120 identified constituents, 21 compounds were absorbed into the bloodstream; phytolaccagenin suppressed Hepa1-6 proliferation, colony formation, and migration, induced apoptosis, ROS accumulation, and lipid peroxidation in vitro, and inhibited Hepa1-6 syngeneic tumor growth in mice without obvious toxicity. In conclusion, PAFFB exerts anti-HCC effects by regulating metabolic reprogramming and inflammatory signaling, and phytolaccagenin represents a candidate bioactive constituent of PAFFB.

## 1. Introduction

Among primary liver cancers, hepatocellular carcinoma (HCC) is the dominant histopathological form, constituting roughly 75–85% of all cases. As a highly prevalent and frequently lethal malignancy, HCC continues to rank among the leading causes of cancer-related death, with both high incidence and mortality. According to the latest data from the National Cancer Center, the number of new cases of primary liver cancer in 2022 was 367,700, ranking 4th among all new cancer cases and 5th in terms of incidence rate, and the incidence rate of primary liver cancer is still on the rise [1,2]. In terms of treatment, surgical resection, liver transplantation, and locoregional therapies remain the mainstay for early-stage HCC; however, a substantial proportion of patients are diagnosed at an unresectable stage, and there remains a considerable need for systemic therapy [3]. Over the past two decades, the systemic treatment landscape for HCC has evolved from the multikinase inhibitor sorafenib [4] to lenvatinib and immune checkpoint inhibitor-based combinations such as atezolizumab plus bevacizumab [5], which have substantially improved the survival of patients with advanced HCC; nevertheless, the prognosis of advanced HCC remains unsatisfactory, and effective, well-tolerated therapeutic strategies are still urgently needed. In recent years, anti-cancer research using Chinese herbal medicines has attracted increasing attention, as herbal medicines represent a rich source of natural products with significant anti-tumor activity, and the screening of active ingredients and pharmacological studies have become a focus of modern Chinese medicine research [6].

*Phytolaccae acinosa*, a traditional Chinese medicine, is derived from the dried roots of *Phytolacca acinosa Roxb*. or *P. americana* L. of the *Phytolaccaceae* family. It has the effects of inducing diuresis to reduce edema, detoxifying and resolving masses, and promoting diuresis, and is mainly used for treating edema, abdominal distension, carbuncles and other symptoms [7]. Modern pharmacological studies have indeed revealed that certain chemical components in *Phytolaccae acinosa* Radix have in vitro anti-tumor activity. For example, studies have shown that these compounds can induce apoptosis, inhibit proliferation and migration of various cancer cell lines (including liver cancer, lung cancer and breast cancer cells) under laboratory conditions [8]. *Phytolacca* fruit is a newly explored medicinal resource. We selected fresh fruits of *Phytolacca acinosa Roxb*. native to Hengyang, China as raw materials, and used the natural symbiotic strains contained in the fresh fruits for fermentation to obtain *Phytolacca acinosa* fruit fermentation broth (PAFFB). This fermentation broth has anti-oxidant, anti-inflammatory, anti-viral, anti-tumor and other effects [9]. Fermentation is a common bioprocessing strategy for medicinal plants that can modify the chemical composition through microbial biotransformation, thereby transforming or enriching active ingredients and affecting their pharmacological efficacy, bioavailability, and toxicity [10]. For example, microbial glycosidases produced during fermentation catalyze the deglycosylation of triterpenoid saponins into less-glycosylated derivatives or their aglycones, and such structural transformation has been shown to enhance the bioactivity of saponins, as exemplified by ginsenoside conversion during ginseng fermentation [11]. Fermentation of botanical drugs has also been reported to reduce toxic side effects and to generate novel bioactive constituents [10]. Consistent with these reported advantages, our UPLC-Q-TOF-MS/MS profiling revealed that PAFFB contains both triterpenoid saponins and their aglycones, which is in line with fermentation-mediated saponin transformation. However, whether fermentation, compared with the unfermented fruit, enhances the anti-HCC activity, reduces the inherent toxicity of Phytolacca constituents, alters the saponin/triterpenoid profile, or simply represents a different formulation has not been systematically established; the present study was therefore designed to characterize the chemical basis and anti-HCC efficacy of PAFFB and to validate its representative bioactive constituents.

In this study, a rat model of hepatocellular carcinoma (HCC) induced by intraperitoneal injection of diethylnitrosamine (DEN), together with normal SD rats, was used, and molecular biology techniques, omics techniques (transcriptomics, proteomics and metabolomics), as well as ultra-high performance liquid chromatography–mass spectrometry (UPLC-Q-TOF-MS/MS) were employed to explore the mechanism of action and main molecular components of PAFFB in exerting anti-hepatocellular carcinoma effects, so as to provide important theoretical guidance and experimental basis for the subsequent development and utilization of PAFFB, the research and development of new anti-tumor drugs and its clinical application.

## 2. Results

### 2.1. Effects of PAFFB on Body Weight, Survival and Body Condition of Hepatocellular Carcinoma Model Rats

**Figure 1:**
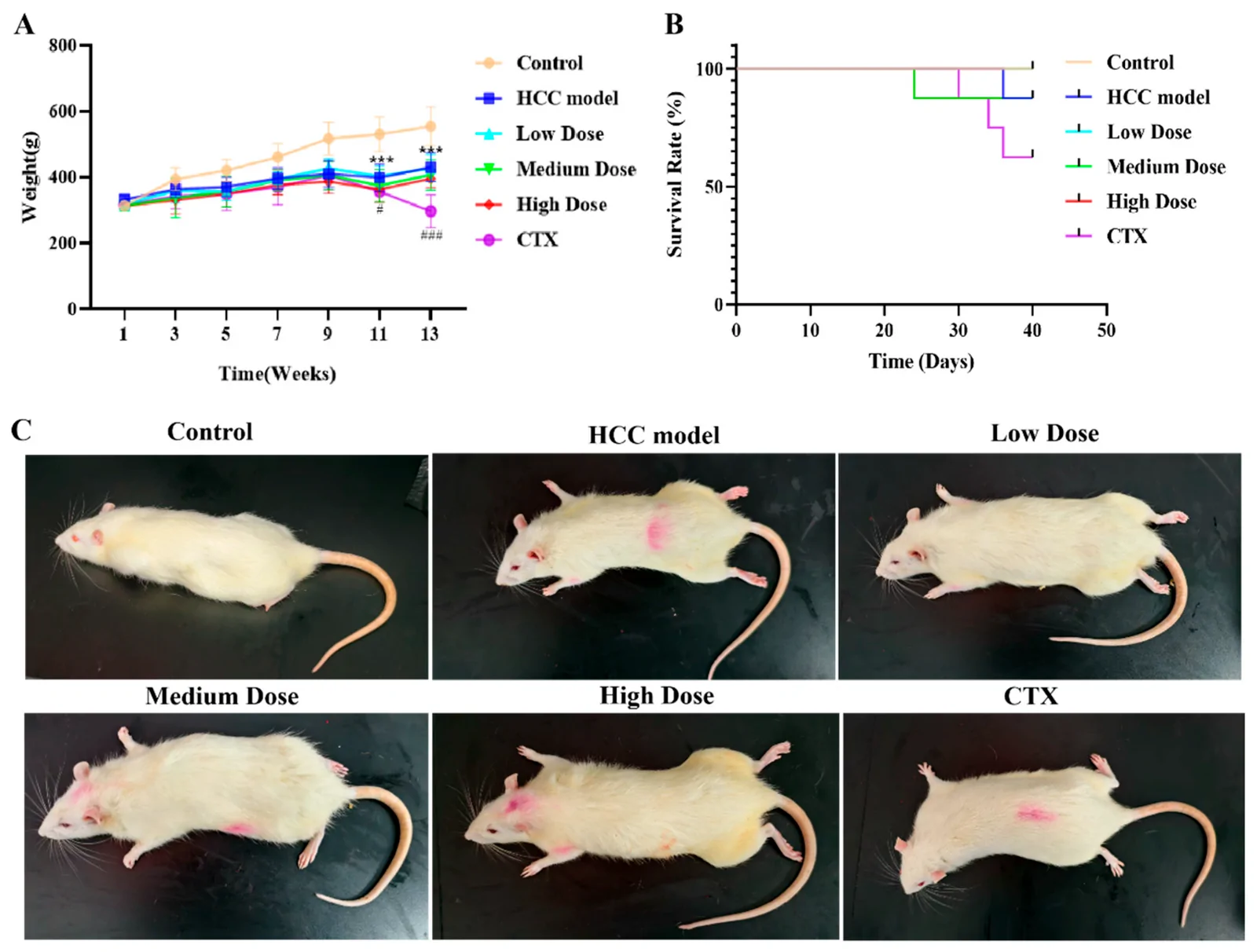
Effects of different doses of PAFFB on body weight, survival status and body condition of HCC model rats. (**A**) Body weight change curve of rats in each group over time; (**B**) Survival curve of rats in each group; (**C**) Observation of general morphology of rats in each group. Data are expressed as the mean ± SD. Comparisons among multiple groups were performed using one-way ANOVA followed by Tukey’s HSD post-hoc test, *n* = 8. *** *p* < 0.001 vs. Control group; # *p* < 0.05, ### *p* < 0.001 vs. HCC model group.

To assess whether PAFFB exerts dose-dependent protection in the DEN-induced HCC rat model, body weight, survival, and gross morphology were monitored in all groups. Weight gain was markedly retarded in model rats relative to controls, which continued to increase steadily. After treatment, the low-, medium-, and high-dose PAFFB groups demonstrated a more favorable weight-gain trend than the model group, whereas CTX administration was associated with a significant decrease in body weight during the late phase (*p* < 0.05, *p* < 0.01; Figure 1A). The survival rate of rats in the HCC model group gradually decreased during the observation period; both the medium dose group and the high dose group could prolong the survival time of rats to a certain extent, but the survival rate of the CTX group was instead reduced. The rats in the control group had a normal body condition and smooth hair (Figure 1B); the HCC model group showed yellowish hair or emaciated body condition, and each administration group had different degrees of improvement, but the rats in the CTX group were more emaciated (Figure 1C). It is suggested that PAFFB has a certain intervention and protective effect on HCC model rats, and this effect is dose-dependent.

### 2.2. The Effect of PAFFB on Liver Morphology and Function in Rats with Hepatocellular Carcinoma

**Figure 2:**
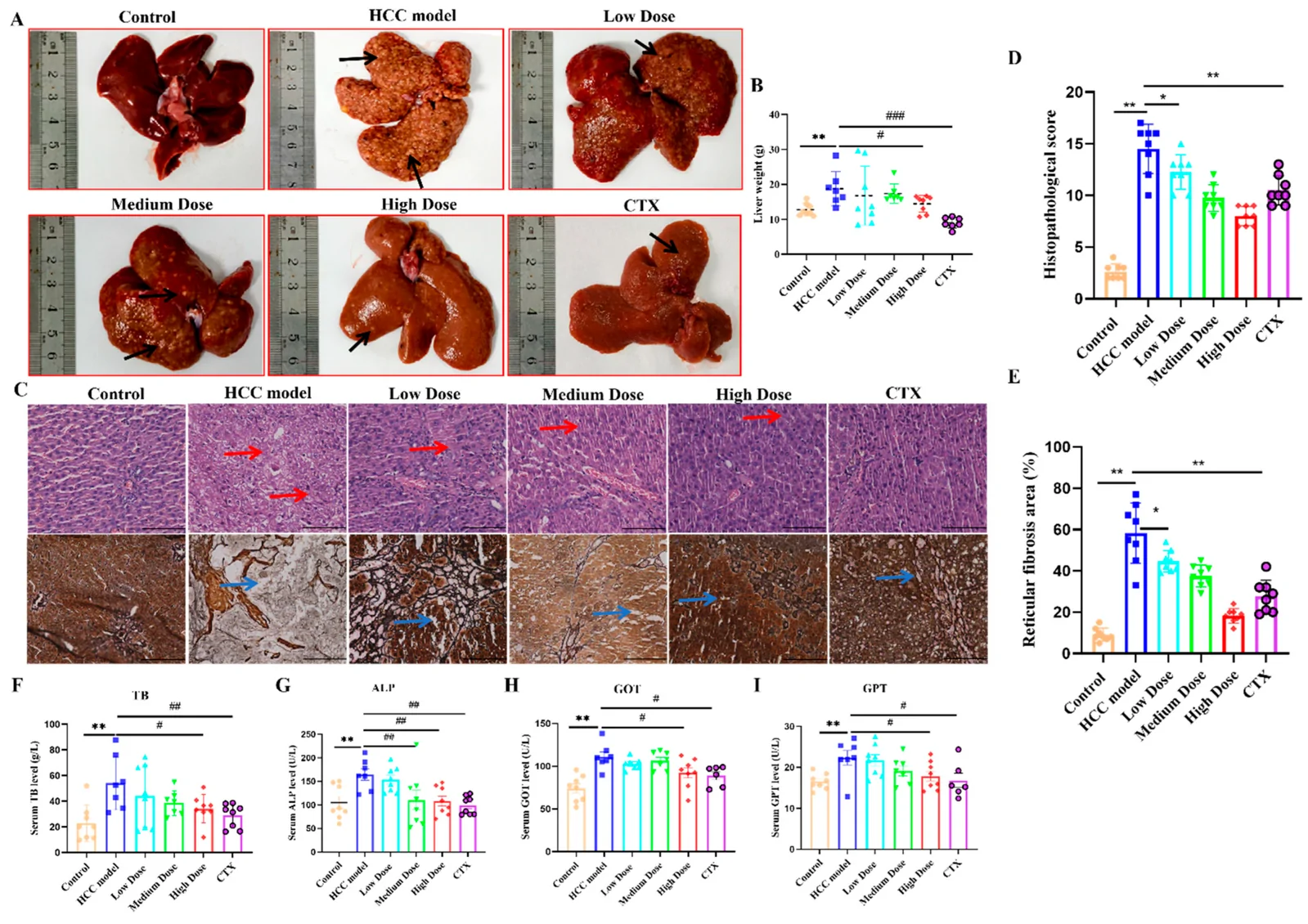
Effects of different doses of PAFFB on liver morphology, weight, pathology, and liver function in HCC model rats. (**A**) Observation of liver morphology and the number of nodules in each group; black arrows indicate macroscopic tumor nodules on the liver surface. (**B**) Quantitative analysis of liver weight in each group. (**C**) Representative images of HE staining and reticulin fibrosis staining (×400) in each group; red arrows indicate cancer nests, blue arrows indicate areas of reticular fibrosis. (**D**) Composite histopathological score (0–20; sum of hepatocellular atypia and cancer nest scores) in each group. (**E**) Quantitative analysis of the reticulin-positive area (%) in each group. (**F**–**I**) Detection of liver function indices in each group, where (**D**) represents total bilirubin (TB), (**E**) represents alanine transaminase (GPT/ALT), (**F**) represents aspartate transaminase (GOT/AST), and (**G**) represents alkaline phosphatase (ALP). Values are presented as means ± SD (*n* = 8 per group). Differences among groups were evaluated by one-way ANOVA with Tukey’s HSD post-hoc correction. ** *p* < 0.01 vs. the control group; * *p* < 0.05, # *p* < 0.05, ## *p* < 0.01, and ### *p* < 0.001 vs. the HCC model group.

Subsequently, we first examined and detected the liver-related parameters in each group of rats, including liver morphology, liver weight, pathological staining, and liver function levels. In the HCC model group, the liver was markedly enlarged relative to controls, and its surface was covered with numerous tumor nodules of varying sizes, accompanied by a significant increase in nodule number (*p* < 0.01). Liver weight was also significantly elevated (*p* < 0.01). HE staining revealed disorganized arrangement of hepatocytes with obvious atypia and the formation of cancer nests, while reticulin fibrosis staining indicated severe hepatic fibrosis. Among the liver function indices, the levels of TB, GPT/ALT, GOT/AST, and ALP were all significantly elevated (*p* < 0.01) (Figure 2), suggesting severe liver injury in the HCC model rats.

In the medium- and high-dose PAFFB groups, liver morphology was substantially improved after treatment: tumor nodule numbers were significantly reduced (Figure 2A), and liver weight returned to near-normal values (*p* < 0.05, *p* < 0.01; Figure 2B). Consistently, HE staining revealed attenuated hepatocellular atypia and fewer cancer nests, while reticulin staining indicated a marked remission of hepatic fibrosis (Figure 2C–E). Furthermore, serum levels of TB, ALT, AST, and ALP declined significantly in a dose-dependent manner (*p* < 0.05, *p* < 0.01; Figure 2F–I). Although the above indices were improved in the low-dose group, no significant differences were observed compared with the HCC model group (*p* > 0.05). The positive drug control (CTX) group significantly reduced the number of tumor nodules and liver function indices; however, it was associated with obvious additional liver tissue damage. Collectively, these results indicate that PAFFB exerts a significant therapeutic and protective effect on liver injury in HCC model rats, and this effect is dose-dependent.

### 2.3. Transcriptomic Analysis

**Figure 3:**
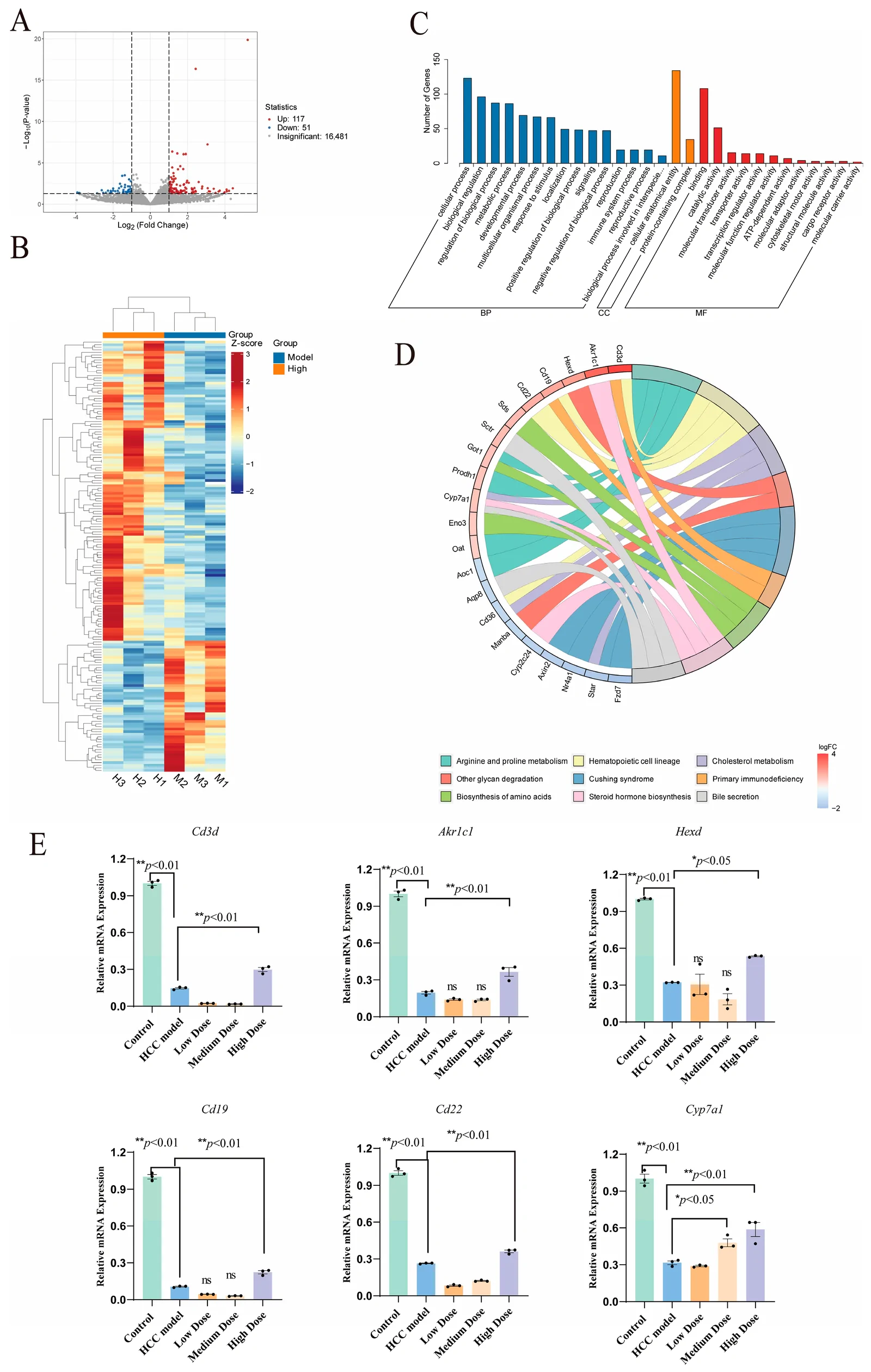
Transcriptomic analysis of PAFFB intervention in DEN-induced hepatocellular carcinoma. (**A**) DEGs volcano map of Model vs. high-dose group. (**B**) Heatmaps of DEGs profile. (**C**) GO term pathways for DEGs. (**D**) KEGG pathway enrichment analysis for DEGs. (**E**) The relative mRNA expression levels of *Cd3d*, *Akr1c1*, *Hexd*, *Cd19*, *Cd22*, and *Cyp7a1* were measured by qRT-PCR. *n* = 3. * *p* < 0.05, ** *p* < 0.01. Total RNA was extracted from the right median lobe of the rat liver, collected at the scheduled sacrifice from the same batch of animals. All values are reported as the mean ± SD. One-way ANOVA, followed by Tukey’s HSD post-hoc test, was used to compare the multiple groups.

Transcriptomic studies offer significant advantages in elucidating the mechanisms of drug action, providing a global perspective for analyzing the impact of drugs on gene expression in cells or tissues [12,13]. By identifying differentially expressed genes (DEGs), researchers can infer the signaling pathways and biological processes potentially targeted by drugs, thereby revealing their molecular mechanisms of action. Based on the aforementioned pharmacological indices, we selected rat liver tissue samples from the model group and the high-dose group for transcriptome sequencing. DEGs were screened using the thresholds of *p* < 0.05 and |log2FoldChange| > 1. As shown in Figure 3A, a total of 168 DEGs were identified compared with the model group, consisting of 51 downregulated and 117 upregulated genes. To visually illustrate the overall changes in the transcriptomic profile of rat liver tissue following intervention with PAFFB, hierarchical clustering analysis was performed on the screened DEGs, and a heatmap was generated. The results, presented in Figure 3B, revealed the following gene expression patterns: after intervention with PAFFB, genes that were aberrantly highly expressed in the high-dose group samples were significantly downregulated.

To further clarify the molecular basis underlying the protective action of PAFFB against DEN-induced hepatocellular carcinogenesis, GO functional enrichment analysis was applied to the DEGs obtained from liver transcriptome profiling. GO categorizes the functional annotations of DEGs into three aspects: biological process (BP), cellular component (CC), and molecular function (MF). The corresponding results are shown in Figure 3C. At the biological process level, the DEGs were primarily enriched in cellular process, biological regulation, metabolic process, and developmental process. Notably, a substantial number of genes were significantly enriched in response to stimulus, signal transduction, and immune system process. This result suggests that PAFFB may inhibit the progression of hepatocellular carcinoma by modulating cellular stress and damage repair, interfering with the activity of signal transduction pathways, and regulating the hepatic immune microenvironment.

The DEGs were then subjected to KEGG pathway enrichment analysis, and the outcomes were visualized using a chord diagram (Figure 3D). In the diagram, the connecting lines illustrate the correspondence between genes and pathways, while node size and color reflect the significance of enrichment. The analysis revealed that the DEGs were significantly enriched in multiple metabolic and signaling pathways closely associated with the occurrence and development of hepatocellular carcinoma, primarily including Arginine and proline metabolism, Biosynthesis of amino acids, Cholesterol metabolism, Steroid hormone biosynthesis, and Bile secretion. The main genes involved included *Cd3d*, *Akr1c1*, *Hexd*, *Cd19*, *Cd22*, and *Cyp7a1*. To further validate these findings, qPCR analysis was performed on several differentially expressed genes identified in the analysis (including *Cd3d*, *Akr1c1*, *Hexd*, *Cd19*, *Cd22*, and *Cyp7a1*), and these findings were in line with the transcriptomic results (Figure 3E).

### 2.4. Proteomics Analysis

**Figure 4:**
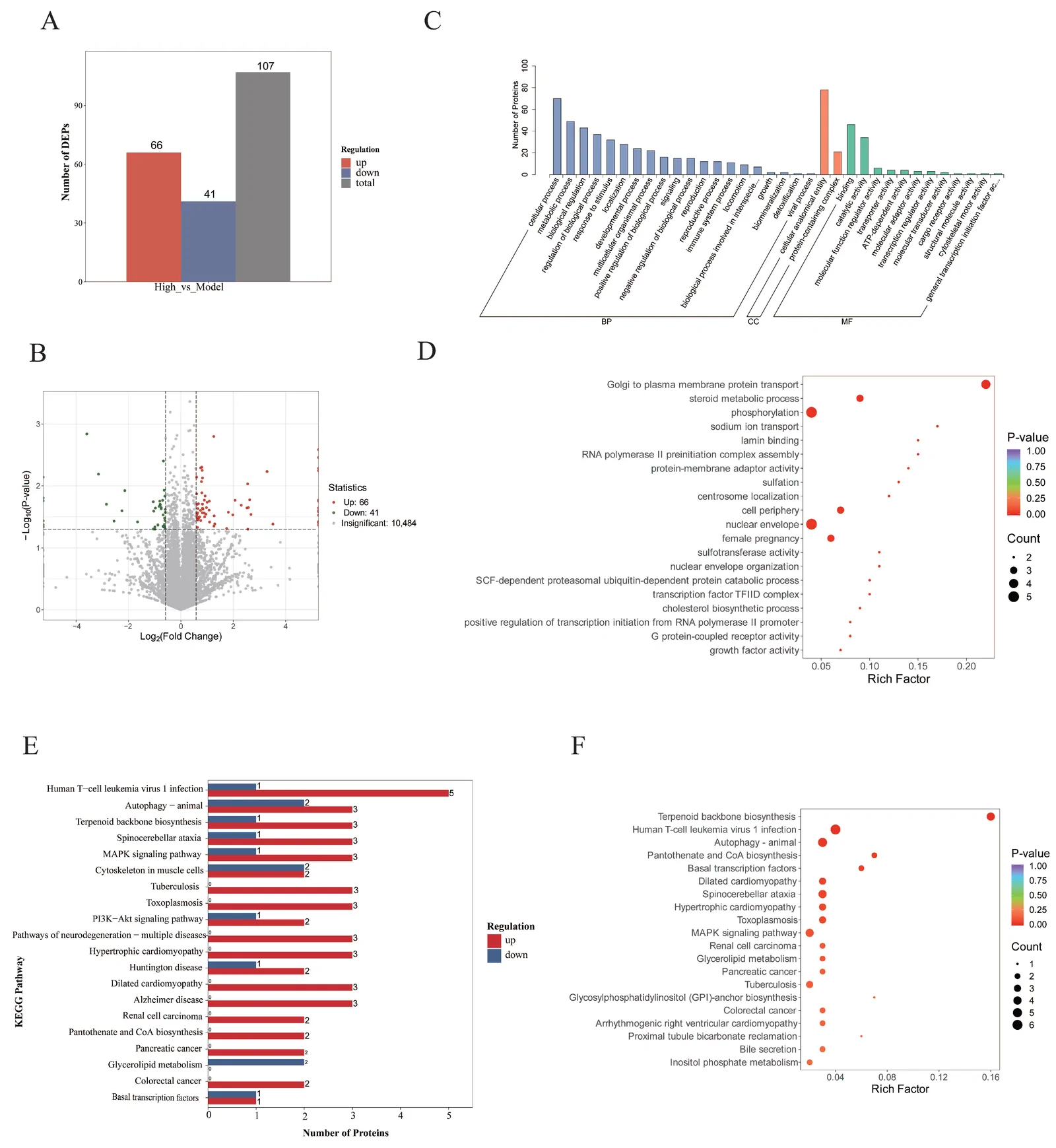
Proteomics analysis of PAFFB intervention in DEN-induced hepatocellular carcinoma. (**A**) Number of differential expression proteins. (**B**) DEPs volcano map of Model vs. High-dose group. (**C**) GO functional enrichment and classification analysis of DEPs. (**D**) GO enrichment analysis of DEPs between High-dose and Model groups. (**E**) KEGG enrichment bar plot of DEPs between High-dose and Model groups. (**F**) KEGG enrichment bubble plot of DEPs between High-dose and Model groups.

DIA quantitative proteomics technology was employed to identify the key proteins targeted by PAFFB. Proteomic profiling of liver tissue specimens led to the identification of 10,591 proteins in total. Using stringent criteria (FC ≥ 1.5 and *p* ≤ 0.05), 107 proteins were screened as differentially expressed between the model group and the high-dose group. Relative to the model group, the treatment group showed 41 downregulated and 66 upregulated proteins (Figure 4A,B). The differentially expressed proteins from liver tissue were then subjected to GO functional enrichment and classification analysis, which covered the three categories of BP, CC, and MF. As shown in the GO classification bar chart (Figure 4C), at the biological process level, these proteins were predominantly enriched in cellular process, metabolic process, and biological regulation, a result consistent with the transcriptomic observation of “multi-gene synergistic regulation”. At the molecular function level, enrichment was mainly observed for catalytic activity and binding. Notably, the pronounced enrichment of proteins related to response to stimulus implies that PAFFB may confer hepatoprotective effects through the modulation of oxidative stress and inflammatory responses.

To gain deeper insight into the molecular mechanisms through which PAFFB exerts its anti-hepatocellular carcinoma activity at the level of protein function execution, GO enrichment analysis and KEGG pathway enrichment analysis were performed on the differentially expressed proteins identified between the high-dose group and the model group (High vs. Model). The results are presented as a GO enrichment analysis bubble chart (Figure 4D) and a KEGG pathway enrichment analysis bar chart (Figure 4E). As shown in Figure 4F, the differentially expressed proteins were widely distributed across multiple tumor-related pathways. Notably, the “Human T-cell leukemia virus 1 infection” pathway contained five up-regulated proteins; this pathway is functionally associated with NF-κB signaling and immune responses. In several oncogenic pathways, including the “PI3K-Akt signaling pathway,” “MAPK signaling pathway,” and “Autophagy-animal,” the differentially expressed proteins were predominantly up-regulated, whereas proteins in some cancer-related pathways, including “Renal cell carcinoma,” “Pancreatic cancer,” and “Colorectal cancer,” were predominantly down-regulated. Of particular interest, all three differentially expressed proteins in the “Terpenoid backbone biosynthesis” pathway were upregulated. This pathway serves as the precursor synthesis route for cholesterol and isoprenoid compounds. Its upregulation corroborates the enrichment of the “Cholesterol metabolism” pathway observed in our transcriptomic analysis, further supporting the hypothesis that PAFFB may exert its anti-hepatocellular carcinoma effects by modulating lipid metabolism.

### 2.5. Metabolomics Analysis

**Figure 5:**
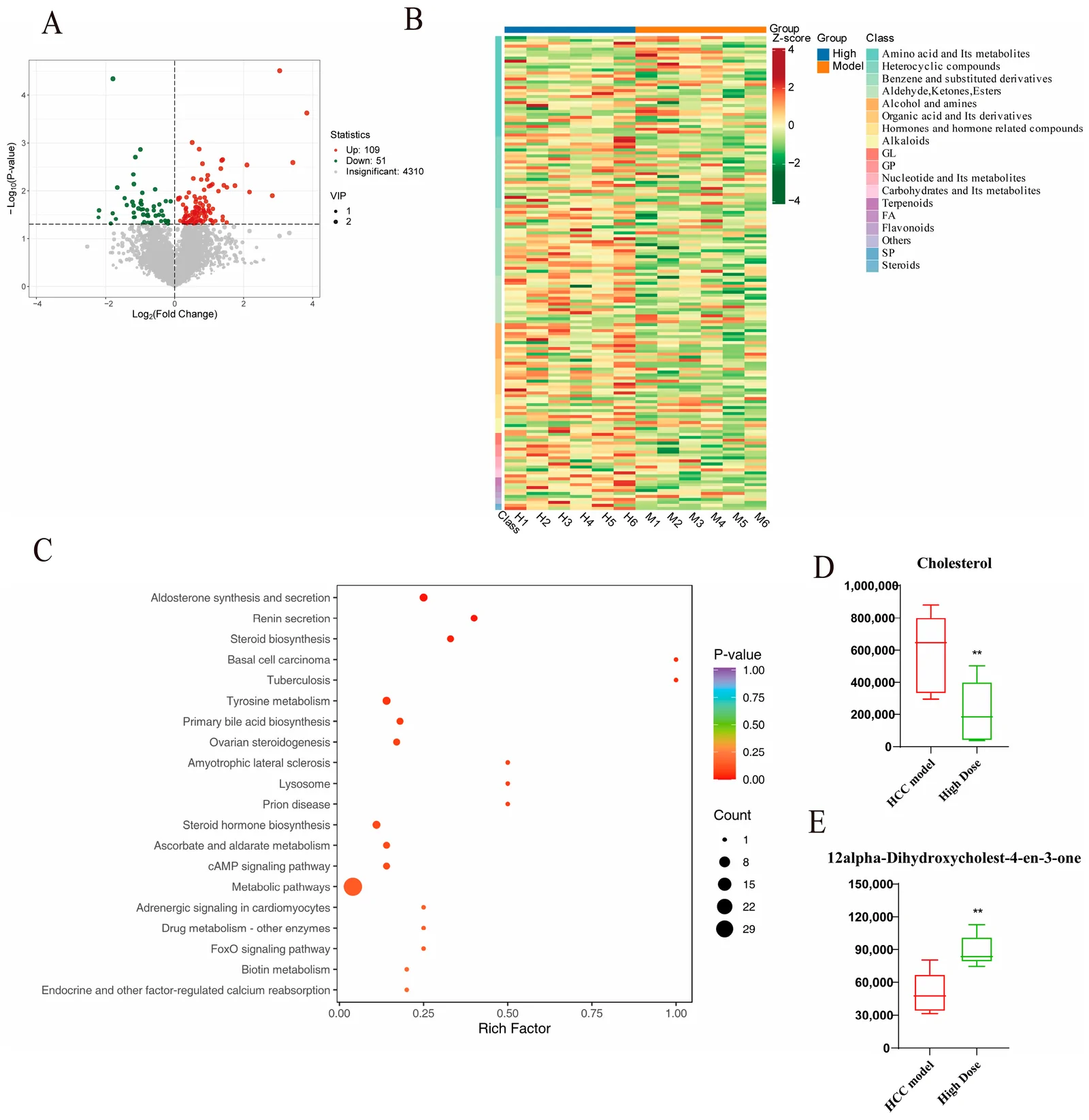
Metabolomics analysis of PAFFB intervention in DEN-induced hepatocellular carcinoma. (**A**) Differential metabolites volcano map of Model vs. High-dose group. (**B**) Heat map of differential metabolites. (**C**) Pathway analysis of differential metabolites. (**D**,**E**) The regulatory effect of PAFFB on the differential metabolites cholesterol and 7α,12α-dihydroxy-4-cholesten-3-one, ** *p* < 0.01. Data are expressed as the mean ± SD. The independent-samples *t*-test was applied for comparisons between two groups (**D**,**E**).

Metabolites satisfying the thresholds of VIP > 1, *p* < 0.05 and |log_2_FC| > 1 were screened, yielding 160 molecules with divergent abundance when comparing the high-dose cohort with the model group. Among these candidates, 109 metabolites showed elevated levels while the remaining 51 exhibited reduced abundance (Figure 5A). These metabolites mainly comprised sphingolipids, bile acids, lipids, steroids, and glycerophospholipids. Heatmap analysis (Figure 5B) revealed that the metabolite levels in the high-dose treatment group were significantly altered relative to the model group, further supporting the positive regulatory role of PAFFB in metabolic modulation.

To further elucidate the anti-hepatocellular carcinoma mechanism of PAFFB at the level of terminal metabolic products, KEGG pathway enrichment analysis was performed on the differential metabolites identified between the high-dose group and the model group. The results are presented as a bubble plot (Figure 5C). As shown, the differential metabolites were significantly enriched in multiple metabolic pathways closely associated with the occurrence and development of hepatocellular carcinoma. Notably, the enrichment of “Primary bile acid biosynthesis” and “Steroid hormone biosynthesis” pathways provided cross-omics corroboration with the transcriptomic findings of “Bile secretion” and “Cholesterol metabolism” as well as the proteomic finding of “Terpenoid backbone biosynthesis.” This indicates that PAFFB not only regulates bile acid and cholesterol metabolism at the gene and protein levels but also directly influences the generation and transformation of bile acids and steroid hormones at the metabolite level. This finding mechanistically accounts for the observed improvements in serum total bilirubin (TBIL) and liver function indices in the animal experiments from the perspective of functional endpoints. Additionally, the enrichment of “Tyrosine metabolism” and “Ascorbate and aldarate metabolism” pathways suggested that PAFFB may alleviate DEN-induced oxidative stress damage by modulating amino acid metabolism and antioxidant metabolism. The enrichment of the “cAMP signaling pathway” corresponded with the regulation of the “PI3K-Akt signaling pathway” observed in the proteomic analysis. As a second messenger, alterations in cAMP metabolism may mediate the cascade of upstream signal transduction. Collectively, these results demonstrate that PAFFB significantly alters the metabolic profile associated with HCC through the coordinated regulation of bile acid metabolism, steroid metabolism, amino acid metabolism, and second messenger metabolic networks at the terminal metabolite level. At the individual metabolite level, two differential metabolites mapped to “Primary bile acid biosynthesis” were identified: compared with the Model group, cholesterol was significantly down-regulated (Figure 5D), whereas 7α,12α-dihydroxy-4-cholesten-3-one, a key intermediate of the classic bile-acid biosynthetic pathway, was significantly up-regulated in the PAFFB-H group (Figure 5E).

### 2.6. Cross-Omics Pathway Consistency Analysis

**Figure 6:**
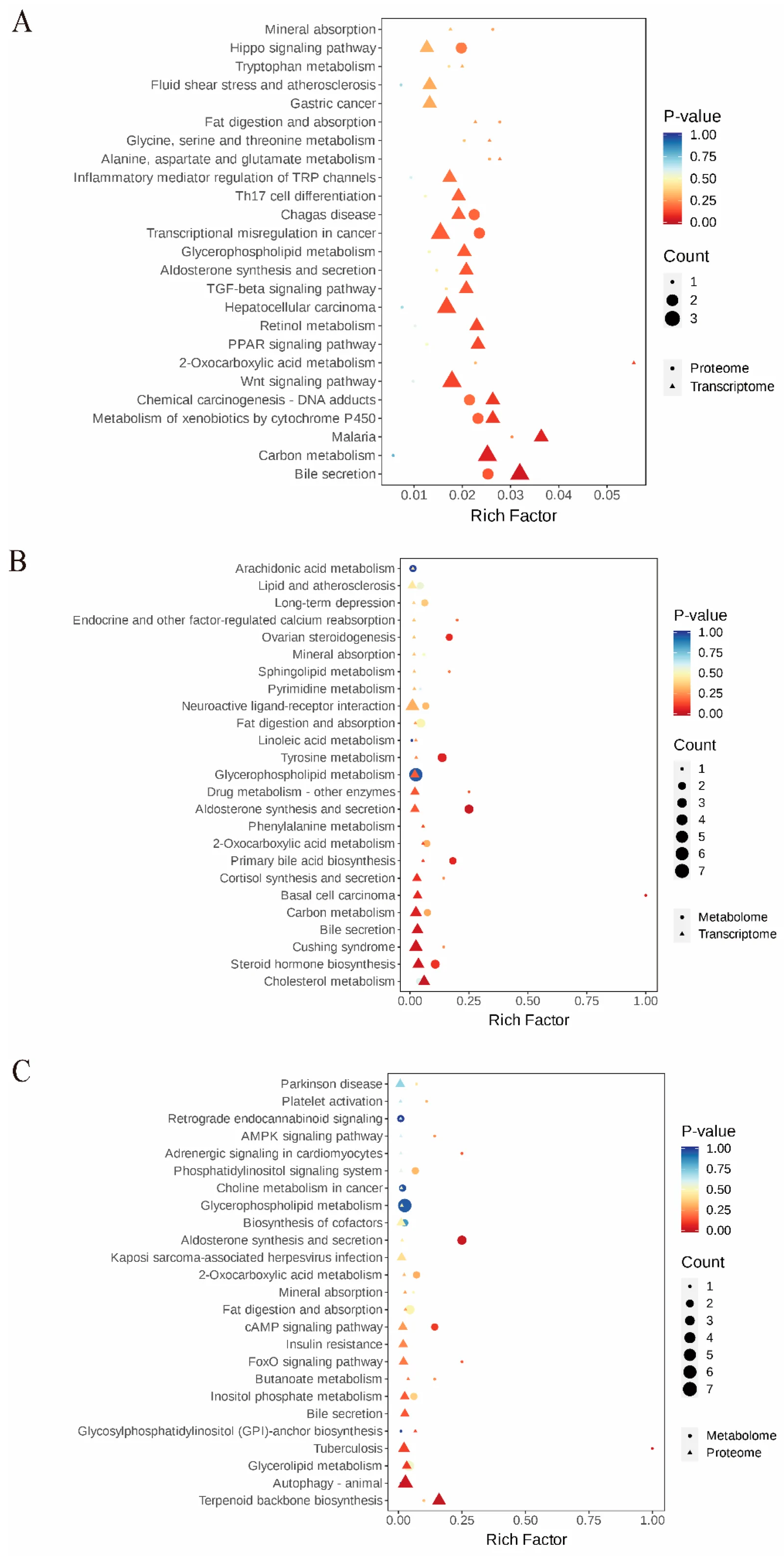
(**A**)Pathways of KEGG enrichment for DEGs and DEPs. (**B**) Pathways of KEGG enrichment for DEGs and DEMs. (**C**) Pathways of KEGG enrichment for DEMs and DEPs.

Transcriptomic-Proteomic Pathway Comparison: To elucidate the anti-hepatocellular carcinoma mechanism of PAFFB from a cross-level perspective integrating gene transcription and protein functional execution, an integrated analysis of transcriptomic and proteomic data was performed to identify KEGG pathways commonly enriched by both omics datasets. The results are presented as a bubble plot (Figure 6A). As shown, the two omics datasets were commonly enriched in multiple signaling and metabolic pathways closely associated with the occurrence and development of hepatocellular carcinoma. These pathways primarily included the Hepatocellular carcinoma pathway, Hippo signaling pathway, Wnt signaling pathway, TGF-beta signaling pathway, Bile secretion, Glycerophospholipid metabolism, Retinol metabolism, Fat digestion and absorption, PPAR signaling pathway, Th17 cell differentiation, Metabolism of xenobiotics by cytochrome P450, and several amino acid metabolism pathways. Collectively, the integrated transcriptomic and proteomic analysis revealed that, relative to the model group, PAFFB treatment was associated with coordinated regulation of multiple DEN-associated pathological pathways at both the gene transcription and protein function levels, including core oncogenic pathways in hepatocellular carcinoma, development-related signaling pathways (Hippo/Wnt/TGF-β), lipid metabolism and bile acid homeostasis, amino acid and energy metabolic reprogramming, the immune-inflammatory microenvironment, and xenobiotic metabolism and detoxification. These commonly enriched pathways constitute the “core regulatory network” underlying the anti-hepatocellular carcinoma effects of PAFFB.

Transcriptomic-Metabolomic Pathway Comparison: To further elucidate the anti-hepatocellular carcinoma mechanism of PAFFB from a cross-level perspective integrating gene expression and terminal metabolic products, an integrated analysis of transcriptomic and metabolomic data was performed to identify KEGG pathways commonly enriched. The results are presented as a bubble plot (Figure 6B). As shown, the two omics datasets were commonly enriched in multiple metabolic and signaling pathways closely associated with the occurrence and development of hepatocellular carcinoma. These pathways primarily included Cholesterol metabolism, Steroid hormone biosynthesis, Primary bile acid biosynthesis, Bile secretion, Glycerophospholipid metabolism, Sphingolipid metabolism, Arachidonic acid metabolism, Linoleic acid metabolism, Tyrosine metabolism, Phenylalanine metabolism, 2-Oxocarboxylic acid metabolism, Neuroactive ligand-receptor interaction, Drug metabolism–other enzymes, and Basal cell carcinoma. Collectively, the integrated transcriptomic and metabolomic analysis revealed that PAFFB was associated with coordinated alterations of multiple pathways at both the gene transcription and protein function levels, including the “cholesterol–bile acid metabolic axis,” phospholipid and polyunsaturated fatty acid metabolism, amino acid and energy metabolism, neuroendocrine signaling, and xenobiotic metabolism and detoxification. Notably, the common enrichment of the Cholesterol metabolism and Primary bile acid biosynthesis pathways across both omics datasets provides a complete chain of evidence from genes to metabolites, forming the core regulatory module underlying the anti-hepatocellular carcinoma effects of PAFFB.

Proteomic-Metabolomic Pathway Comparison: To elucidate the anti-hepatocellular carcinoma mechanism of PAFFB from a cross-level perspective integrating protein functional execution and terminal metabolic products, an integrated analysis of proteomic and metabolomic data was performed to identify KEGG pathways commonly enriched by both omics datasets. The results are presented as a bubble plot (Figure 6C). As shown, the two omics datasets were commonly enriched in multiple signaling and metabolic pathways closely associated with the occurrence and development of hepatocellular carcinoma. These pathways mainly comprised the AMPK, cAMP, and FoxO signaling pathways, together with bile secretion, glycerophospholipid metabolism, glycerolipid metabolism, fat digestion and absorption, and butanoate metabolism. Taken together, the combined proteomic and metabolomic analyses indicated that, compared with the model group, PAFFB administration was accompanied by coordinated modulation of several DEN-related pathological pathways at both the protein function and metabolite levels, involving the energy-sensing network (AMPK/cAMP/FoxO), lipid metabolism, and bile acid homeostasis.

### 2.7. Effects of PAFFB on Inflammatory Signaling Pathways and Key Metabolic Enzyme Expression in the Liver of HCC Rats

Multi-omics analyses repeatedly indicated the enrichment of inflammation- and immunity-related pathways, with NF-κB and MAPK serving as central hubs in inflammatory signal transduction. Toll-like receptor 4 (TLR-4), a pattern recognition receptor, recognizes damage-associated molecular patterns (DAMPs) and activates downstream NF-κB (p65) and MAPK (p38) pathways via the adaptor protein MYD88, mediating the release of inflammatory cytokines [14,15]. Accordingly, we evaluated the expression levels of TLR-4, MYD88, total p65, p-p65, total p38, and p-p38 to validate the regulatory effects of PAFFB on the hepatic inflammatory microenvironment. Furthermore, multi-omics analyses consistently pointed to the regulation of the cholesterol–bile acid metabolic axis, in which CYP7A1 acts as the classic rate-limiting enzyme in bile acid synthesis, catalyzing the conversion of cholesterol to 7α-hydroxycholesterol—the first committed step in the bile acid biosynthetic pathway [16,17]. Changes in the expression of this enzyme directly determine the flux of bile acid synthesis. Therefore, we measured the protein expression level of CYP7A1 to assess the regulatory effects of PAFFB on bile acid synthesis. CYP4B1, a member of the cytochrome P450 family, is involved in fatty acid hydroxylation (particularly in arachidonic acid metabolism) and xenobiotic metabolism [18]. The enrichment of drug metabolism—other enzymes and arachidonic acid metabolism pathways in the multi-omics analyses suggested that PAFFB may affect the expression of P450 enzymes. Hence, we also examined the protein level of CYP4B1 to explore the regulatory effects of the drug on hepatic detoxification metabolism and lipid peroxidation pathways.

**Figure 7:**
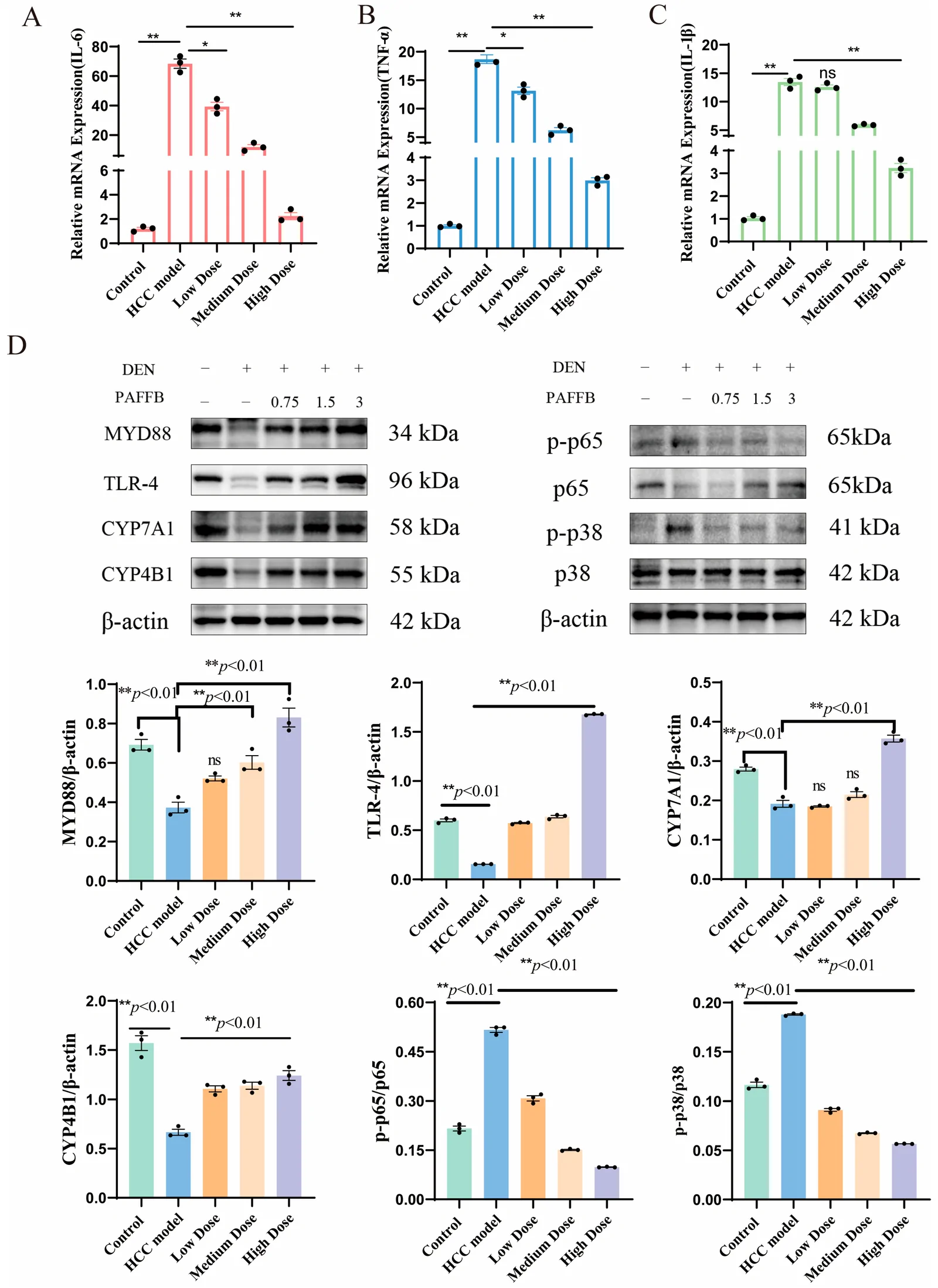
(**A**–**C**) The relative mRNA expression levels of IL-6, TNF-α, and IL-1β in rat liver tissue, measured by qRT-PCR (*n* = 3). (**D**) The level of protein expression in liver tissue was detected by Western blot. * *p* < 0.05, ** *p* < 0.01. All values are reported as the mean ± SD. One-way ANOVA, followed by Tukey’s HSD post-hoc test, was used to compare the multiple groups.

As presented in Figure 7, hepatic mRNA abundances of the NF-κB-driven pro-inflammatory cytokines IL-6, TNF-α and IL-1β were markedly upregulated in the Model cohort in contrast to the control cohort (*p* < 0.01), which reflects heightened downstream inflammatory signaling within the tumor-bearing liver. Compared with this elevated baseline, PAFFB intervention brought about a pronounced suppression of the aforementioned cytokine transcripts (*p* < 0.01), as displayed in panels A–C of Figure 7.

When evaluated at the protein-expression axis, hepatic abundance of TLR-4 and MYD88 in the model cohort declined sharply relative to the untreated controls (*p* < 0.01). In contrast, the phosphorylation ratios p-p65/p65 and p-p38/p38 rose markedly under the same comparison (*p* < 0.01). Meanwhile, the protein expression of CYP7A1 and CYP4B1 was significantly downregulated (*p* < 0.01). Following intervention with the high-dose PAFFB group, compared with the model group, the expression of TLR-4 and MYD88 was significantly restored (*p* < 0.01), the ratios of p-p65/p65 and p-p38/p38 were significantly decreased (*p* < 0.01), and the protein expression of CYP7A1 and CYP4B1 was significantly upregulated (*p* < 0.01) (Figure 7D). These findings indicate that PAFFB exerts its anti-hepatocellular carcinoma effects by systematically improving the immunosuppressive state, excessive activation of inflammatory signaling, and disruption of bile acid metabolism in the DEN-induced hepatocellular carcinoma model at the protein function level. This is achieved through restoring the basal expression levels of the TLR-4/MYD88 axis, inhibiting the phosphorylation activation of p65 and p38, and upregulating the expression of the rate-limiting enzyme CYP7A1 in bile acid synthesis. Notably, the “decoupling phenomenon” characterized by decreased TLR-4/ MYD88 expression concomitant with increased phosphorylation of downstream p65/p38 in the model group suggests the presence of TLR-independent inflammatory signaling activation pathways in advanced hepatocellular carcinoma (e.g., oxidative stress, TNF-α autocrine signaling, or direct activation by bile acids) [19,20]. In contrast, intervention with PAFFB simultaneously targeted both upstream receptor expression and downstream kinase activity, demonstrating its multi-target synergistic regulatory characteristics.

### 2.8. The Effect of PAFFB on Lipid Metabolism-Related Indicators in the Liver of HCC Model Rats

**Figure 8:**
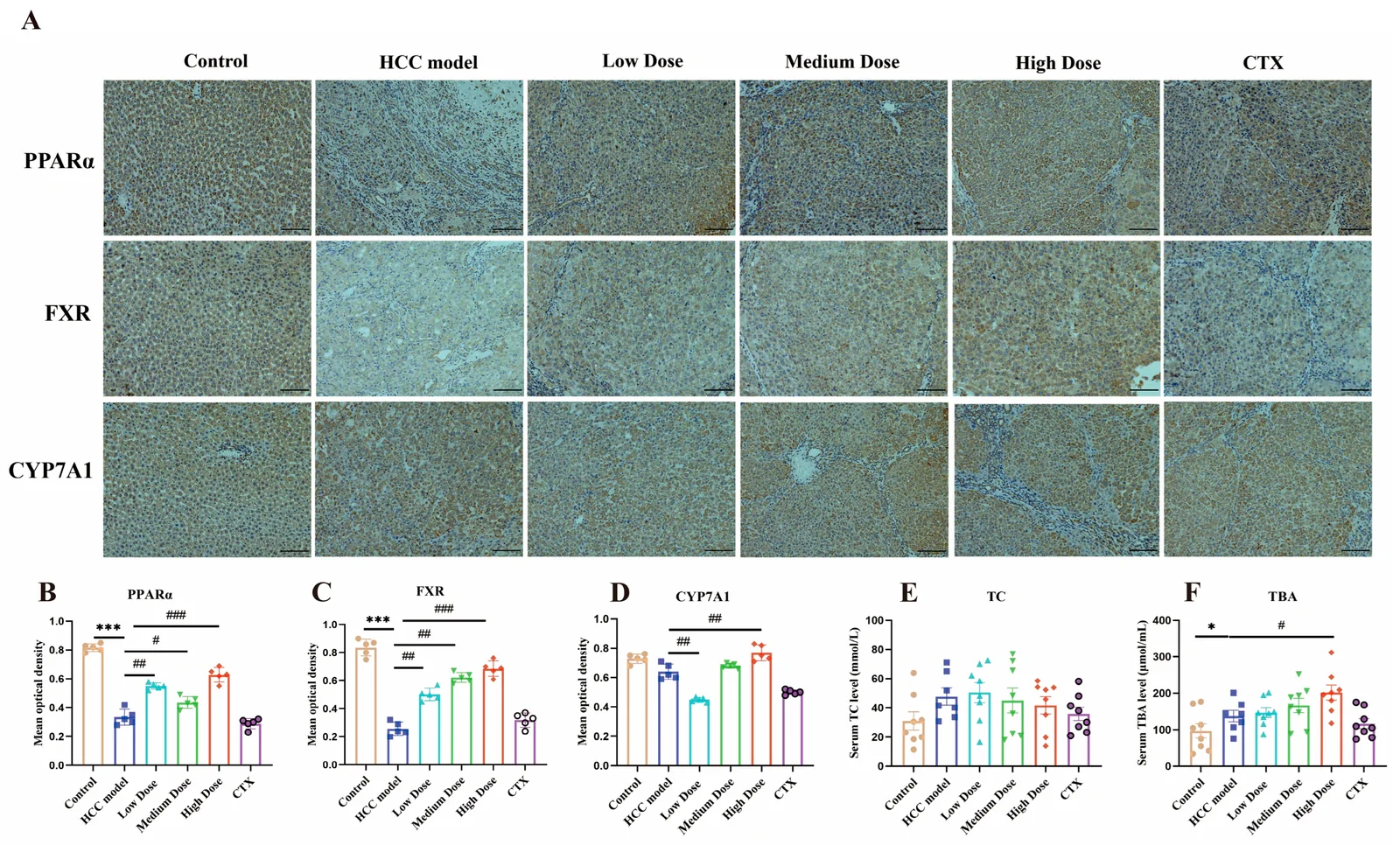
Effects of PAFFB on indicators related to hepatic lipid metabolism in HCC model rats. (**A**) Representative immunohistochemical images of PPARα, FXR, and CYP7A1 in the liver of rats in each group (scale bar = 50 μm); (**B**–**D**) Quantitative analysis of PPARα (**B**), FXR (**C**), and CYP7A1 (**D**) protein expression in the liver (*n* = 5); (**E**,**F**) Determination of serum total cholesterol (TC, (**E**)) and total bile acid (TBA, (**F**)) levels in rats of each group (*n* = 8). Values are presented as the mean ± SD. One-way ANOVA with Tukey’s HSD post-hoc test was used for comparisons involving multiple groups. * *p* < 0.05 and *** *p* < 0.001 vs. the control group; # *p* < 0.05, ## *p* < 0.01, and ### *p* < 0.001 vs. the HCC model group.

Building upon the preceding omics evidence, our investigation narrowed to the central regulatory circuits governing hepatic lipid turnover and bile-acid balance, and examined whether PAFFB modulates the PPARα–FXR–CYP7A1 axis. Immunohistochemical staining demonstrated that, in contrast to controls, hepatic PPARα and FXR protein abundance in the HCC model cohort was markedly reduced (*p* < 0.001). By contrast, CYP7A1—the pivotal rate-limiting enzyme of bile-acid biosynthesis—was likewise suppressed (Figure 8A–D), which points to disrupted transcriptional control of hepatic metabolic programs in the model animals. Following PAFFB administration, the protein expression of PPARα and FXR in the liver of the medium- and high-dose groups was significantly increased in a dose-dependent manner (*p* < 0.05, *p* < 0.01) (Figure 8B,C), and the expression level of CYP7A1 was also effectively restored (*p* < 0.01) (Figure 8D). Correspondingly, serological detection showed that the serum TC of rats in the HCC model group had an increasing trend, and the TBA level was significantly increased (*p* < 0.05). The PAFFB treatment group could reduce the TC level and significantly increase the serum TBA level (*p* < 0.05) (Figure 8E,F). In conclusion, PAFFB may activate the PPARα/FXR signaling pathway, up-regulate the expression of CYP7A1, thereby improving the hepatic lipid metabolism disorder of HCC rats, promoting bile acid synthesis, and exerting a protective effect on the hepatic metabolic microenvironment.

### 2.9. Characterization of PAFFB’s Chemical Profile and Blood-Absorbed Components

**Figure 9:**
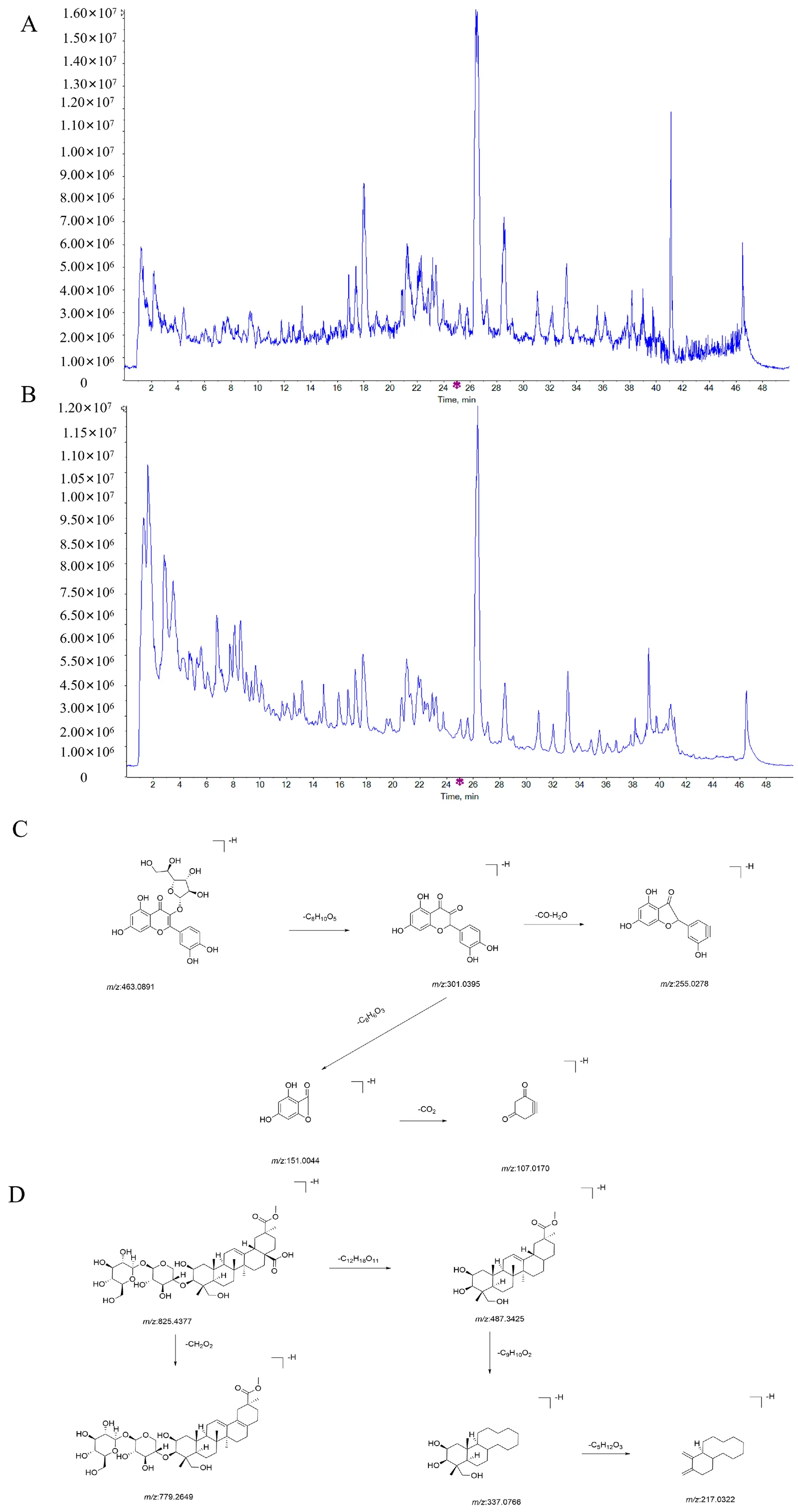
Identification of the components of PAFFB. (**A**,**B**) Total ion chromatograms of the in vitro components of PAFFB; acquired in negative and positive ion modes. (**C**) Mass spectrometric fragmentation pathways of quercetin 3-O-β-D-glucofuranoside. (**D**) Mass spectrometric fragmentation pathways of Esculentoside A.

With the dual aims of clarifying the constituent profile of PAFFB and exploring its candidate anti-hepatocellular carcinoma activity, UPLC-Q-TOF-MS/MS was adopted to characterize both its full chemical composition and the components detectable in circulating blood. Panels A and B of Figure 9 display the resulting total ion chromatograms (TICs) acquired in negative- and positive-ion modes, respectively, on an electrospray ionization (ESI) source. By comparing retention times, parent ions, and fragment ions with reference standards and databases, including GNPS: https://gnps.ucsd.edu/, (accessed on 17 July 2025), ChemSpider: http://www.chemspider.com/ (accessed on 6 August 2025), and MassBank: https://massbank.jp/MassBank/ (accessed on 8 August 2025), a total of 120 compounds were identified (Table 1) [21,22].

Given that this plant abounds in flavonoids and triterpenoids, compounds **63** and **97** were selected as representative examples to propose the plausible mass spectrometric fragmentation routes for these two compound classes. Compound **63** (quercetin 3-O-β-D-glucofuranoside), a flavonoid eluting at 12.62 min, displayed a quasi-molecular ion at *m*/*z* 463.0891 [M−H]^−^, corresponding to the molecular formula C_21_H_20_O_12_, and harbors a glucofuranosyl moiety. Under negative ion mode, cleavage of the glucofuranosyl group gave rise to the fragment at *m*/*z* 301.0395 [M-H-C_6_H_10_O_5_]^−^. Further dehydration together with the loss of a carbonyl group afforded the ion at *m*/*z* 255.0278 [M-H-C_6_H_10_O_5_-H_2_O-CO]^−^. In parallel, retro-Diels-Alder (RDA) cleavage produced the fragment ion at *m*/*z* 151.0044 [M-H-C_6_H_10_O_5_-C_8_H_6_O_3_]^−^, which underwent further loss of an ester group to furnish the fragment at *m*/*z* 107.0170 [M-H-C_6_H_10_O_5_-C_8_H_6_O_3_-CO_2_]^−^. Compound **63** was thus assigned as quercetin 3-O-β-D-glucofuranoside, and its proposed fragmentation pathway is depicted in Figure 9C. Compound **97** exhibited a retention time of 25.15 min, with a quasi-molecular ion peak at *m*/*z* 825.4377 [M−H]^−^. Decarboxylation followed by reduction yielded the fragment ion at *m*/*z* 779.2649 [M-H-CH_2_O_2_]^−^. Alternatively, the loss of two sugar moieties generated the fragment at *m*/*z* 487.3425 [M-H-C_12_H_18_O_11_]^−^. Subsequent ring cleavages produced the ion fragments at *m*/*z* 337.0766 [M-H-C_12_H_18_O_11_-C_9_H_10_O_2_]^−^ and *m*/*z* 217.0322 [M-H-C_12_H_18_O_11_-C_9_H_10_O_2_-C_5_H_12_O_3_]^−^. Compound **97** was definitively confirmed as Esculentoside A, and its proposed fragmentation pathway is shown in Figure 9D.

**Figure 10:**
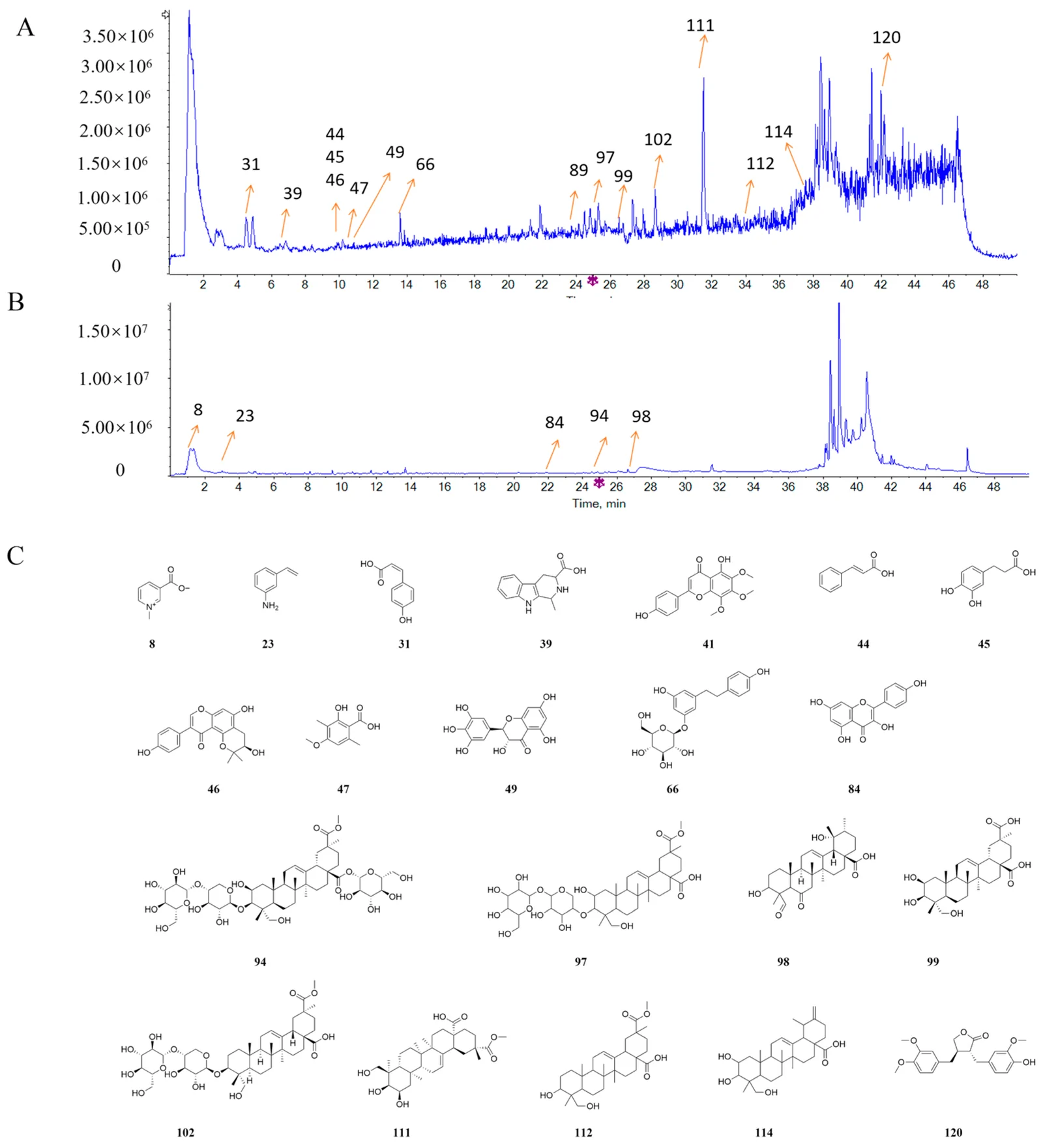
Identification of the components absorbed into blood. (**A**,**B**) Total ion chromatograms of drug-containing plasma acquired in negative and positive ion modes. (**C**) The 21 Blood-Absorbed active components of PAFFB.

To pinpoint constituents reaching systemic circulation, the TICs of blank plasma were contrasted against those acquired from plasma harvested after oral dosing with PAFFB. This side-by-side comparison uncovered a cluster of chromatographic signals appearing solely in the post-dose specimens, which points to successful intestinal uptake of certain PAFFB-origin compounds (Figure 10A,B). Through structural annotation of these circulating constituents, 21 bioactive molecules were recognized as having entered the bloodstream (Figure 10C). They fell into multiple chemical families, chiefly phenolic acids (e.g., trans-cinnamic acid), flavonoids (e.g., ampelopsin and kaempferol), and triterpenoids (e.g., esculentoside A and esculentoside H). Firm characterization of these plasma-accessible compounds offers key evidence for the likely direct mediators underlying PAFFB’s in vivo anti-hepatocellular carcinoma activity, and in turn lays a reliable groundwork for dissecting its molecular mechanism.

### 2.10. Phytolaccagenin Inhibits Hepa1-6 Cell Growth and Migration and Promotes ROS Accumulation and Lipid Peroxidation

Our preliminary UPLC-Q-TOF-MS/MS analysis identified 21 compounds absorbed into the bloodstream following PAFFB administration, among which Phytolaccagenin—a characteristic triterpenoid aglycone of Phytolacca species—drew our attention due to its reported pharmacological activities. However, whether this specific compound directly contributes to the anti-HCC efficacy of PAFFB remains unknown. Therefore, in the present study, we further investigated the anti-hepatocellular carcinoma effects of Phytolaccagenin both in vitro and in vivo, aiming to validate whether this absorbed component serves as one of the direct pharmacological mediators of PAFFB’s therapeutic action.

**Figure 11:**
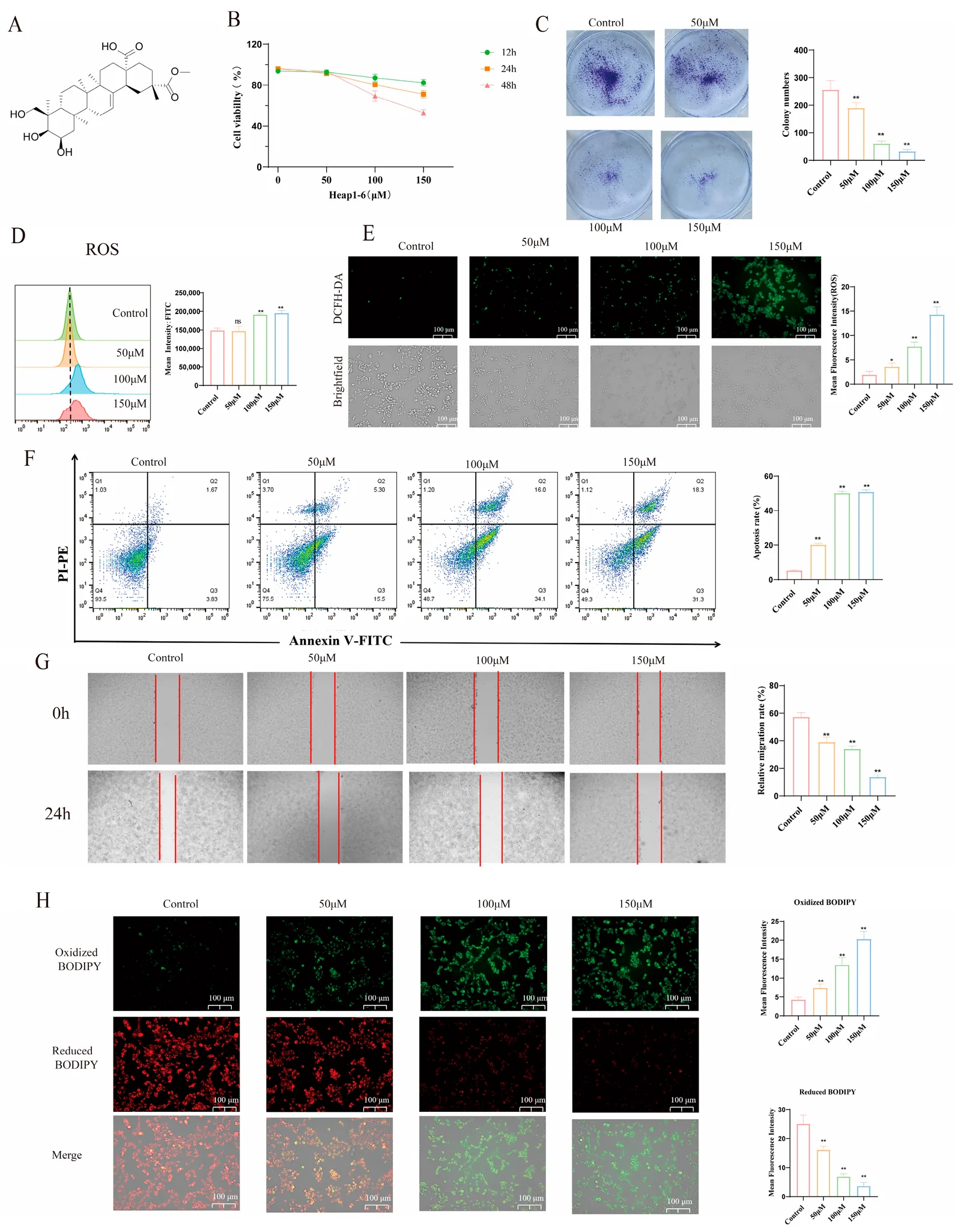
Phytolaccagenin inhibits Hepa1-6 cell proliferation and migration, and induces apoptosis, ROS accumulation, and lipid peroxidation in vitro. (**A**) Chemical structure of Phytolaccagenin. (**B**) Cell viability of Hepa1-6 cells treated with increasing concentrations (50, 100, and 150 μM) of Phytolaccagenin for 12, 24, and 48 h, as determined by CCK-8 assay. (**C**) Representative images and quantitative analysis of colony formation assays showing the number and area of colonies after Phytolaccagenin treatment. (**D**,**E**) Intracellular reactive oxygen species (ROS) levels detected by flow cytometry (**D**) and immunofluorescence staining with DCFH-DA probe (**E**). (**F**) Apoptosis rates evaluated by Annexin V-FITC/PI double staining flow cytometry. (**G**) Wound healing assay showing cell migration at 0 and 24 h after Phytolaccagenin treatment. (**H**) Lipid peroxidation levels assessed by C11-BODIPY immunofluorescence, depicting the shift from reduced (red) to oxidized (green) probe; * *p* < 0.05, ** *p* < 0.01. All values are reported as the mean ± SD. One-way ANOVA, followed by Tukey’s HSD post-hoc test, was used to compare the multiple groups.

To this end, we first examined the direct effects of Phytolaccagenin (chemical structure shown in Figure 11A) on Hepa1-6 cells. As presented in Figure 11B, Phytolaccagenin treatment significantly suppressed cell viability in a dose- and time-dependent manner across concentrations of 50, 100, and 150 μM. The colony formation assay further revealed that Phytolaccagenin markedly reduced both the number and the area of Hepa1-6 colonies, indicating a potent inhibition of long-term proliferative capacity (Figure 11C). Concurrently, both flow cytometry (Figure 11D) and immunofluorescence staining (Figure 11E) revealed a marked elevation of intracellular ROS levels following Phytolaccagenin treatment. Flow cytometric analysis with Annexin V-FITC/PI staining showed that Phytolaccagenin significantly increased the apoptotic cell population in a concentration-dependent manner (Figure 11F). In addition, the wound healing assay demonstrated that Phytolaccagenin treatment for 24 h dose-dependently delayed scratch closure, suggesting an inhibitory effect on cell migration (Figure 11G). As shown in Figure 11H, Phytolaccagenin treatment induced a pronounced shift of the C11-BODIPY probe from the reduced to the oxidized state, confirming the accumulation of lipid peroxidation products in a dose-dependent manner. Collectively, phytolaccagenin inhibits Hepa1-6 cell proliferation, colony formation, and migration and induces apoptosis in vitro, effects associated with ROS accumulation and lipid peroxidation, supporting it as a direct pharmacological mediator of the anti-HCC activity of PAFFB.

### 2.11. Phytolaccagenin Suppresses Tumor Growth in a Hepa1-6 Syngeneic Tumor Mouse Model

**Figure 12:**
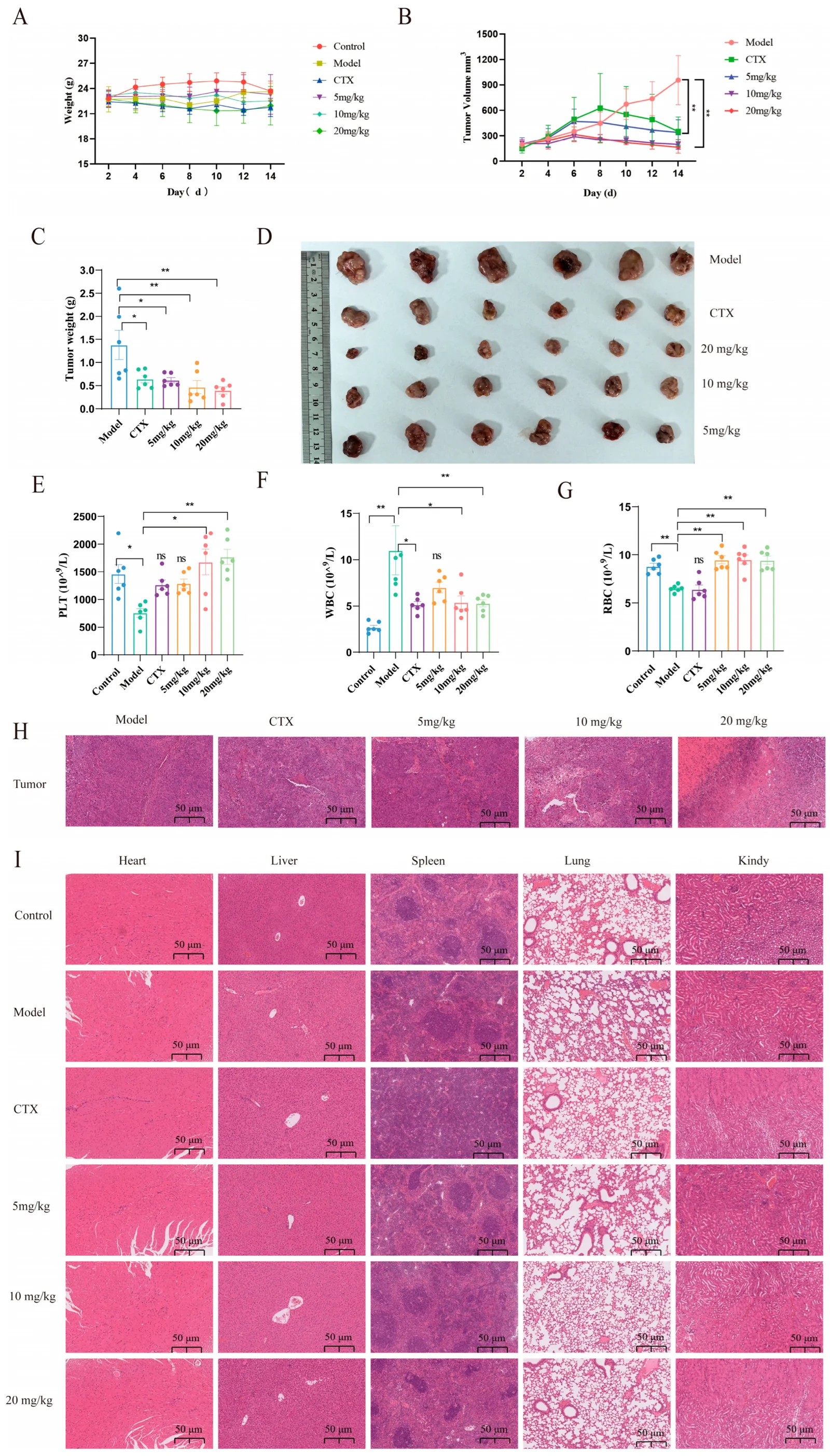
Phytolaccagenin suppresses Hepa1-6 syngeneic subcutaneous tumor growth in C57BL/6 mice with a favorable safety profile. (**A**) Variation in body mass across the 14-day dosing window. (**B**) Growth trajectories of tumor volume, recorded at 2-day intervals. (**C**) Final tumor mass harvested at experiment termination. (**D**) Photographic comparison of excised tumors from every cohort. (**E**) Circulating platelet (PLT) counts in peripheral blood. (**F**) Leukocyte, i.e., white blood cell (WBC), counts. (**G**) Erythrocyte, i.e., red blood cell (RBC), counts. (**H**) H&E-stained sections of tumor tissue. (**I**) H&E staining of representative vital organs, covering the heart, liver, spleen, lung and kidney. Quantitative data are presented as mean ± standard deviation; pairwise comparisons across groups were resolved by one-way analysis of variance (ANOVA) coupled with Tukey’s HSD post-hoc test (*n* = 6 per cohort). * *p* < 0.05, ** *p* < 0.01.

To further corroborate the in vitro findings and evaluate the therapeutic potential of Phytolaccagenin in vivo, we established a Hepa1-6 subcutaneous xenograft mouse model. As shown in Figure 12A, treatment with Phytolaccagenin at doses of 5, 10, and 20 mg/kg did not cause significant body weight loss throughout the observation period, indicating the lower systemic toxicity of Phytolaccagenin. In terms of anti-tumor efficacy, Phytolaccagenin treatment dose-dependently suppressed the growth of Hepa1-6 subcutaneous tumors. As presented in Figure 12B, the tumor volume in the Phytolaccagenin-treated groups increased more slowly than that in the model group throughout the 14-day treatment period, with the 20 mg/kg group showing the most pronounced inhibition. Consistently, tumor weight at the end of the experiment was significantly reduced in a dose-dependent manner (Figure 12C), and representative tumor images (Figure 12D) visually confirmed the smaller size of tumors in the Phytolaccagenin groups compared with the model group. CTX, although also effective in reducing tumor burden, caused notable weight loss and poor general condition in mice.

To assess the hematopoietic safety of Phytolaccagenin, we measured peripheral blood platelet (PLT), white blood cell (WBC), and red blood cell (RBC) counts in each group. As illustrated in Figure 12E–G, there were no significant differences in PLT, WBC, or RBC levels between the control group and any of the Phytolaccagenin-treated groups. In contrast, the CTX group exhibited a reduction in RBC and PLT counts, reflecting myelosuppression—a common adverse effect of conventional chemotherapeutic agents. Histopathological examination further supported the safety and efficacy of Phytolaccagenin. H&E staining of tumor tissues (Figure 12H) revealed extensive necrosis, increased nuclear pyknosis, and reduced cellularity in the Phytolaccagenin-treated groups compared with the model group, confirming its tumor-suppressive activity. More importantly, H&E staining of major organs—including the heart, liver, spleen, lung, and kidney—showed no obvious pathological damage or morphological abnormalities in any of the Phytolaccagenin-treated groups, whereas the CTX group exhibited mild hepatic sinusoid congestion and splenic atrophy (Figure 12I). Collectively, these in vivo results demonstrate that Phytolaccagenin effectively inhibits Hepa1-6 syngeneic tumor growth without causing significant toxicity in the parameters assessed, which is consistent with the in vitro observations and supports its role as a safe and active anti-HCC component derived from PAFFB.

## 3. Discussion

In this study, we employed an integrative multi-omics strategy combining transcriptomics, proteomics, and metabolomics, alongside pharmacodynamic evaluation and chemical profiling, to elucidate the anti-HCC mechanism of PAFFB. Our results demonstrate that PAFFB exerts its therapeutic effects by coordinately modulating the cholesterol–bile acid metabolic axis and the TLR-4/MYD88/NF-κB/MAPK inflammatory signaling pathway, thereby systematically modulating the metabolic reprogramming and immunosuppressive microenvironment associated with HCC. Consistent with our previous in vitro findings, PAFFB treatment dose-dependently improved body weight loss, survival rate, and general condition in DEN-induced HCC rats. Macroscopically, PAFFB significantly reduced liver weight and the number of tumor nodules, while histopathological examination revealed restoration of hepatic lobular architecture and attenuation of reticular fibrosis. Notably, with respect to the parameters assessed in this study, PAFFB showed less apparent toxicity than the positive control cyclophosphamide.

Hepatocellular carcinoma remains a leading cause of cancer-related death worldwide, and a substantial proportion of patients are diagnosed at an unresectable stage, leaving systemic therapy as the mainstay of treatment [3]. Although multi-kinase inhibitors such as sorafenib and lenvatinib, and the immune-checkpoint-inhibitor combination atezolizumab plus bevacizumab, have improved survival outcomes, their efficacy is limited by primary and acquired resistance and by treatment-related toxicities [4,5]. Multi-target natural products with favorable tolerability therefore remain an important source of adjunctive therapeutic candidates for HCC [6]. Consistent with this rationale, PAFFB dose-dependently improved survival, body weight, and liver function and reduced tumor burden in the DEN-induced HCC rat model, while exhibiting less apparent toxicity than cyclophosphamide in the parameters assessed.

A striking feature of our multi-omics analysis was the consistent enrichment of pathways related to cholesterol metabolism, bile secretion, and steroid hormone biosynthesis across all three omics layers. This convergence strongly implicates the cholesterol–bile acid metabolic axis as a central target of PAFFB. Mechanistically, PAFFB treatment restored the expression of PPARα and FXR, two master regulators of lipid homeostasis, and subsequently upregulated CYP7A1, the rate-limiting enzyme in the classic bile acid synthesis pathway. This transcriptional and translational recovery was corroborated by reduced serum total cholesterol and increased total bile acid levels, indicating enhanced cholesterol catabolism. Given that CYP7A1 downregulation and FXR suppression are hallmarks of HCC-associated metabolic dysfunction, our data suggest that PAFFB exerts its anti-HCC effects, at least in part, by reactivating this axis. Aberrant bile-acid homeostasis and FXR dysfunction are increasingly recognized as drivers of hepatocarcinogenesis: FXR expression is downregulated in HCC patient tissues and is associated with a worse prognosis, and FXR-deficient mice develop spontaneous HCC accompanied by elevated bile-acid levels [23]. Our observation that PAFFB restored the PPARα/FXR/CYP7A1 axis and shifted serum cholesterol toward bile-acid synthesis is therefore consistent with the tumor-suppressive roles of FXR and CYP7A1-mediated cholesterol catabolism reported previously [17]. To our knowledge, reactivation of the classic bile-acid biosynthetic pathway by a fermented herbal preparation, as evidenced here by the reciprocal changes in cholesterol and 7α,12α-dihydroxy-4-cholesten-3-one, has rarely been reported. Beyond metabolic regulation, pathway enrichment analysis revealed significant involvement of immune-related pathways, including Th17 differentiation, NF-κB, and MAPK signaling. Notably, we observed a “decoupling phenomenon” in the HCC model group: decreased TLR-4/MYD88 protein expression alongside elevated phosphorylation of downstream p65/p38. This correlative observation raises the hypothesis that TLR-4-independent inflammatory signaling pathways may contribute to NF-κB/MAPK activation in advanced hepatocellular carcinoma. Potential drivers reported in previous literature include oxidative stress, TNF-α autocrine signaling [24], and direct activation triggered by accumulated bile acids [16,19]. However, direct molecular evidence for TLR-4-independent activation is absent in the present study, and this interpretation remains speculative.

To bridge the chemical profile with pharmacological activity, we characterized the components of PAFFB and identified 21 absorbed compounds in rat plasma, including flavonoids (e.g., quercetin glycosides, kaempferol), phenolic acids, and triterpenoids (e.g., esculentoside A). Notably, several of these compounds have been previously reported to modulate PPARγ/FXR signaling or exhibit anti-inflammatory properties [25]. Among these blood-absorbed components, Phytolaccagenin—a characteristic triterpenoid aglycone of Phytolacca species—drew our attention as a potential direct mediator of PAFFB’s anti-HCC efficacy. It should be noted that a recent study reported hepatotoxicity of phytolaccagenin in zebrafish and LO2 cells mediated by ferroptosis and PPAR/P53 signaling [26]. Although no obvious toxicity was observed in the present mouse study at the tested doses, this report underscores the importance of systematic safety evaluation of Phytolacca-derived constituents and supports our conservative interpretation of the safety profile of PAFFB. To validate this hypothesis, we systematically evaluated its pharmacological activity both in vitro and in vivo. Our results demonstrated that Phytolaccagenin dose-dependently inhibited Hepa1-6 cell viability, colony formation, and migration, while promoting apoptosis, ROS accumulation, and lipid peroxidation—a phenotypic profile that closely mirrors the oxidative stress-related pathways identified in our multi-omics analysis of PAFFB. More importantly, in a Hepa1-6 syngeneic C57BL/6 mouse model, Phytolaccagenin administration significantly suppressed tumor growth without causing body weight loss or hematopoietic toxicity, recapitulating the favorable safety profile observed with PAFFB. Collectively, these findings establish Phytolaccagenin as one of the bona fide active principles of PAFFB, providing direct experimental evidence that links the chemical profile of PAFFB to its pharmacological activity.

Limitations of the present study should also be acknowledged. PAFFB is a complex spontaneous fermentation broth. Although untargeted UPLC-Q-TOF-MS/MS profiling tentatively identified 120 chemical constituents, the current work only achieved qualitative and relative-abundance profiling. Owing to the limited commercial availability of authentic reference standards for most triterpenoid-glycoside ingredients, absolute concentrations for major components as well as phytolaccagenin in PAFFB have not been determined. Future work will develop targeted quantitative LC-MS assays for marker compounds to support quality-control standardization of PAFFB. In the current study, purified phytolaccagenin standard was administered at well-defined dosages for independent pharmacological validation, rather than inferring activity from its endogenous concentration within the crude fermentation broth. Furthermore, a direct comparative analysis between the fermented broth and the unfermented fruit was not performed in this study; whether fermentation enhances the anti-HCC activity of PAFFB, reduces the inherent toxicity of Phytolacca constituents, or simply represents a different formulation therefore remains to be clarified, and the specific contribution of fermentation to chemical transformation, bioactivity, and toxicity warrants dedicated investigation in future studies.

In addition, dedicated pharmacokinetic parameters (Cmax, AUC, t_1_/_2_) of phytolaccagenin after oral PAFFB administration have not been characterized in the current study. Specific in-vivo anti-tumor references for phytolaccagenin are scarce. The 5–20 mg/kg dosage of purified phytolaccagenin applied in mouse tumor models was determined based on our in-vitro cellular activity results and preliminary animal tolerance tests. This dosage range produced no obvious toxicity in C57BL/6 mice and was used to verify its intrinsic anti-HCC bioactivity. Importantly, this dose was not calibrated to match the actual systemic exposure of phytolaccagenin generated by PAFFB in rats. PAFFB exerts its anti-HCC effect via multiple blood-absorbed components in combination, and phytolaccagenin represents only one among 21 absorbed ingredients. Future pharmacokinetic studies will be required to clarify the in-vivo exposure level of phytolaccagenin derived from PAFFB and construct reliable dose-exposure-effect relationships.

In conclusion, this integrative multi-omics study demonstrates that PAFFB exerts its anti-HCC effects through coordinated regulation of the cholesterol–bile acid metabolic axis and the TLR-4/MYD88/NF-κB/MAPK inflammatory pathway. Furthermore, the identification and functional validation of Phytolaccagenin as a representative blood-absorbed active component bridge the chemical profile of PAFFB to its pharmacological efficacy, reinforcing the “from whole to single component” research paradigm in traditional Chinese medicine. These findings not only highlight the therapeutic potential of PAFFB as a multi-target agent for HCC treatment but also provide a lead compound for further drug development. Our work provides a solid foundation for further development of PAFFB and its active constituents as promising adjuvant therapeutic agents for hepatocellular carcinoma.

## 4. Materials and Methods

### 4.1. Chemicals and Reagents

DEN (A919866-10) and Cyclophosphamide (CXT) (A545946-3) was obtained from AmBeed Company (Arlington Heights, IL, USA). Serum alanine transaminase (GPT/ALT) assay kit (AMHD3-M96, Nanjing, China), aspartate transaminase (GOT/AST) assay kit (AMHD2-M96, Nanjing, China), alkaline phosphatase (ALP) activity assay kit (AMHD4-M96, Nanjing, China), total bilirubin (TB) assay kit (AYHF5-M96, Nanjing, China), total cholesterol (TC) content assay kit (AYHC1-M96, Nanjing, China), and total bile acid (TBA) content assay kit (AMHG1-M96, Nanjing, China) were purchased from Nanjing Mofan Biotechnology Co., Ltd. (Nanjing, China). Antibody against GAPDH (DGR11217) was obtained from Diagbio Biotechnology Co., Ltd. (Hangzhou, China). Antibody against Cytochrome P450 7A1(CYP7A1) (DF2612) was purchased from Affinity Biosciences (Cincinnati, OH, USA). Antibodies against PPARα (P010554) and FXR (R014984) were acquired from Epizyme Biomedical Technology Co., Ltd. (Shanghai, China). Phytolaccagenin (CAS:1802-12-6, Lot No. AFFD2804, purity 98.58%, determined by HPLC peak-area normalization) was obtained from Chengdu Alfa Biotechnology Co., Ltd. (Chengdu, China). Other chemicals and biological reagents were of analytical grade.

### 4.2. Preparation of PAFFB

PAFFB was prepared from fresh fruits of *Phytolacca acinosa Roxb*. by a multi-stage spontaneous fermentation process that required no heating and was performed under light-protected conditions. First, a fermentation seed broth was prepared by naturally fermenting high-quality *Phytolacca acinosa Roxb* fruits, with the culture conditions adjusted by the addition of 0.1–0.5% ammonium phosphate and brown sugar. Fresh ripe fruits were then washed, drained, and air-dried in the shade to 60–70% dryness, placed in non-metallic fermentation vessels, and supplemented with 2–3% of the seed broth together with an appropriate amount of ammonium phosphate and brown sugar aqueous solution. The mixture was fermented at 20–35 °C for 30–60 days, and the fermentation liquid was collected by separation or pressing (first fermentation liquid). The first fermentation liquid was then transferred to non-metallic containers and subjected to a second fermentation at 5–28 °C for another 30–60 days under sealed, light-protected conditions with static sedimentation; the supernatant was collected as the second fermentation liquid. Potassium sorbate was added as an antioxidant, and the liquid was aged in sealed ceramic jars (250 kg or 350 kg capacity) at 8–25 °C in a cool, dark place until an aromatic odor developed. The settled stock was finally adjusted with citric acid and acesulfame potassium, filled into dark, light-protected pharmaceutical bottles, and packaged to obtain the final PAFFB preparation.

### 4.3. Animals and Treatment

A total of 48 male Sprague Dawley (SD) rats, 7 weeks of age and weighing 200 ± 20 g, were obtained from Guangzhou Vital River Laboratory Animal Technology Co., Ltd. The animals were housed under specific pathogen-free (SPF) conditions (22 ± 2 °C, 50 ± 5% relative humidity, 12 h/12 h light/dark cycle) with unrestricted access to standard chow and water, and were allowed to acclimatize for 1 week. The experimental protocol received approval from the Committee on Ethics of Animal Experiments at the Affiliated Nanhua Hospital, University of South China (permit number: 2024-ky-119). All animal procedures complied with the guidelines established by the Chinese Animal Welfare Committee. Two batches of rats were used in this study, both housed, induced with DEN, and treated under identical conditions and in accordance with the same animal ethics protocol. Batch 1 (48 rats; 6 groups, *n* = 8 per group) was used for the longitudinal monitoring of survival. Batch 2 (48 rats; 6 groups, *n* = 8 per group) was used for the terminal measurements at the scheduled endpoint, including body weight, liver morphology and weight, histopathological examination, serum liver-function parameters, and omics analyses. After acclimatization, except the control group, the remaining rats were intraperitoneally injected with DEN at 70 mg/kg once weekly for 12 consecutive weeks to establish an HCC model; normal and PAFFB-treated HCC model rats were randomly divided into 6 groups (*n* = 8 each): Control, HCC model, PAFFB Low/Medium/High dose, and cyclophosphamide (CTX) positive control groups. From the 9th week of DEN injection, rats were intragastrically administered (ig) once daily for 4 weeks: PAFFB (3, 1.5, 0.75 g/kg/d for High, Medium, Low dose groups), CTX (0.07 g/kg, administered every other day), and equal volume of normal saline for Control and HCC model groups. The doses of PAFFB (0.75, 1.5, and 3 g/kg/d) were selected on the basis of preliminary dose-finding experiments.

Following dosing, the animals underwent a 12 h fasting period with water provided ad libitum. At study termination, rats were anesthetized via intraperitoneal injection of sodium pentobarbital (50 mg/kg); urine was harvested, spun at 3000 r/min under 4 °C for 10 min, and the resulting supernatants were kept at −80 °C pending analysis. Prior to euthanasia, DEN-exposed animals showed markedly raised serum ALT/AST levels, which further corroborated successful model induction [27,28]. Animals were then euthanized by cervical dislocation; liver specimens were excised, rinsed in ice-cold normal saline and patted dry. A portion was immersed in 4% paraformaldehyde for histological sectioning, while the remainder was snap-frozen in liquid nitrogen and held at −80 °C for downstream assay of biochemical indices.

### 4.4. Histological Examination

Immediately after euthanasia, each liver was rapidly excised, freed of surrounding connective tissue and adipose tissue, and immersed overnight in 4% paraformaldehyde maintained at 4 °C. The fixed specimens then passed through a stepwise ethanol dehydration protocol (70–100%, 15 min per grade), were cleared in xylene on two successive 10 min rounds, and were paraffin-embedded at 60 °C. Consecutive sections of 5 μm thickness were cut on a Leica RM2245 microtome (Leica Biosystems, Wetzlar, Germany), affixed to poly-L-lysine-coated slides, and subjected to hematoxylin-eosin (HE) staining. One section out of every five was retained, examined and imaged with a Keyence BZ-X800LE microscope (Keyence, Tokyo, Japan) at 100× and 400× magnification. The structure and cell morphology of the liver lobules were observed with Keyence BZ-X800LE Photo Imaging System (Keyence, Tokyo, Japan). Three independent measurements per section were averaged for accuracy. H&E-stained sections were scored semi-quantitatively in a blinded manner for hepatocellular atypia (0–10) and the area of cancer nests (0–10), and the two scores were summed to generate a composite histopathological score (0–20).

### 4.5. Liver Reticular Fibrosis Staining

After sacrifice, rat livers were quickly dissected, trimmed of connective tissue and fat, and fixed in 4% paraformaldehyde at 4 °C overnight. Fixed tissues were dehydrated in graded ethanol (70–100%), cleared in xylene, embedded in paraffin, and sectioned into 5 μm serial slices mounted on poly-L-lysine-coated slides. Reticular fibrosis was assessed by reticular fiber silver staining: sections were sequentially oxidized with 1% periodic acid, impregnated with silver ammonia solution, reduced with 10% formaldehyde, fixed with sodium thiosulfate, and counterstained with nuclear fast red, yielding black/dark brown reticular fibers, red collagen fibers, and pale yellow hepatocytes [29]. Every fifth section was examined and photographed with a Keyence BZ-X800LE microscope (Keyence, Tokyo, Japan) at 100× and 200× magnification. The distribution and proliferation of reticular fibers, as well as the integrity of the hepatic lobular reticular framework, were carefully evaluated. The reticulin-positive area (%) was quantified in five randomly selected fields per section using ImageJ with a fixed threshold applied identically to all sections.

### 4.6. Immunohistochemistry (IHC) Measurements

The streptavidin–peroxidase (SP) conjugated technique (SP Immunohistochemistry Kit, ZSGB-BIO, Beijing, China) was adopted to assay hepatic expression of peroxisome proliferator-activated receptor alpha (PPARα), farnesoid X receptor (FXR), and cholesterol 7α-hydroxylase (CYP7A1) [30,31]. Paraffin blocks were cut into 5 μm sections, deparaffinized in xylene for two 10 min cycles, and rehydrated through a descending ethanol series (100–70%, 5 min per grade). Antigen exposure was achieved by warming the slices in 0.01 M sodium citrate buffer (pH 6.0) at 700 W for 15 min, after which they were allowed to cool naturally. Endogenous peroxidase activity was quenched with 3% hydrogen peroxide (10 min at room temperature), and non-specific sites were sealed with 5% sheep serum (37 °C, 30 min). Overnight incubation at 4 °C used the following primary antibodies: anti-PPARα (1:100; Epizyme P010554, Shanghai, China), anti-FXR (1:200; Epizyme R014984, Shanghai, China), and anti-CYP7A1 (1:200; Biosciences DF2612, Ohio, USA), with PBS substituting as the negative control. After three PBS rinses (5 min each), sections were incubated with biotin-labeled secondary antibody (1:200, ZSGB-BIO) at 37 °C for 30 min, washed again, and incubated with streptavidin–peroxidase conjugate (37 °C, 30 min). Color development was done with DAB substrate, terminated with distilled water. Sections were counterstained with hematoxylin, dehydrated, cleared, and mounted. At least five random non-overlapping fields per section were observed and photographed under an Olympus AH-2 microscope (400×). Olympus CellSens Standard software BX63 quantified positive staining IOD to evaluate PPARα, FXR, and CYP7A1 expression.

### 4.7. Liver Function Indicators Detection

The collected blood was injected into anticoagulant-free vacuum blood collection tubes and allowed to stand at room temperature for 30 min. Then, centrifugation was performed at 3000 r/min for 10 min to separate the upper serum. The detection of serum liver function-related indicators was carried out in strict accordance with the operating procedures specified in the corresponding kit instructions. The specific operations were as follows: an appropriate amount of serum samples and the matching reagents of each kit were taken separately, parameters such as reaction temperature and reaction time were set according to the requirements of the kit instructions, detection was performed on the corresponding microplate reader or biochemical analyzer, the absorbance value or activity value of each indicator was read, and the activity of GPT/ALT, GOT/AST, ALP as well as the concentration of TB were calculated according to the standard curve or calculation formula of the kit.

### 4.8. Serum Total Cholesterol and Total Bile Acid Detection

The detection was performed by enzyme cycling method and micro-method, strictly following the instructions of the TC (AYHCI-M96) and TBA (AMHG1-M96) content detection kits. Serum samples were directly determined. The microplate reader was preheated for more than 30 min, the wavelength was adjusted to 546 nm/405 nm, and zero adjustment was performed with distilled water. In a 96-well plate, reagent 1 and samples (or standard products, distilled water) were added as required, reacted at 37 °C for 5 min, and the absorbance A1 was read; then reagent 2 was added, reacted at 37 °C for 10 min, and the absorbance A2 was read. The absorbance difference was calculated, and the concentration of TC/TBA in the samples was calculated according to the standard tube concentration, the absorbance difference and the corresponding formula.

### 4.9. RNA-Seq Analysis

Six hepatic specimens were processed for total-RNA isolation: three biological replicates from the DEN-induced HCC Model cohort (M1–M3) and three from the PAFFB High-dose cohort (H1–H3). Sample concentration was quantified on a Qubit 4.0 fluorometer, while integrity was evaluated with a Qsep400 bioanalyzer; only RNA passing these quality checks entered library preparation. mRNA was pulled down from total RNA through oligo(dT) magnetic beads and sheared into short pieces; random hexamer primers then guided first-strand cDNA synthesis, with second-strand synthesis performed afterward. The double-stranded cDNA underwent end repair, A-tailing, adapter ligation, purification, PCR amplification, denaturation, and circularization to generate the sequencing library. Library acceptance rested on two checks: insert-size inspection on an Agilent 2100 and effective-concentration quantification by Q-PCR, with a cutoff of effective concentration above 2 nM. Passing libraries were converted into DNA nanoballs (DNBs) and sequenced on the MGI DNBSEQ platform, yielding paired-end 150 bp (PE150) reads. Raw data were trimmed with fastp (v0.23.2), which discarded adapter-bearing reads, N-containing reads and low-quality sequences; this yielded 46.04 Gb of clean data (≥6 Gb per sample) at Q30 ≥ 95%. Clean reads were mapped to the Rattus norvegicus reference genome (Ensembl, mRatBN7.2.109) via HISAT2 (v2.2.1) under default settings. Gene-level counts were generated with featureCounts (v2.0.3), and expression was expressed as FPKM (fragments per kilobase of transcript per million fragments mapped). Sample reproducibility was assessed through principal-component analysis alongside sample-correlation analysis. Differential expression between the Model and High-dose cohorts was called by DESeq2 (v1.38.3) on unnormalized count matrices; resulting *p*-values underwent Benjamini–Hochberg correction, and a gene was declared a DEG when *p* < 0.05 and |log2FoldChange| > 1. To clarify the biological roles carried by the DEGs, downstream functional annotation combined two complementary strategies: over-representation analysis based on the hypergeometric test across GO (biological process, cellular component and molecular function), KEGG pathways and Reactome, together with gene set enrichment analysis (GSEA) run against the same GO, KEGG and Reactome gene-set collections [32].

### 4.10. Proteomic Analysis

In this project, quantitative DIA proteomics technology was employed. First, proteins were extracted, enzymatically digested, and desalted from six rat liver samples (3 biological replicates for the Model group (M1-M3) and 3 biological replicates for the High-dose group (H1-H3)). In brief, frozen liver specimens were pulverized to a fine powder in liquid nitrogen and disrupted in lysis buffer composed of 8 M urea, 1 mM PMSF and 2 mM EDTA, with sonication carried out on ice. After centrifugation, the BCA kit was used to quantify protein yield. For each sample, 100 μg of protein was reduced with 5 mM DTT (37 °C, 45 min), alkylated with 11 mM iodoacetamide (room temperature, darkness, 15 min), and then digested overnight with trypsin (Promega). Resulting peptides were desalted over C18 resin.

Peptides were separated on a Vanquish Neo UHPLC nano-LC system. Loading used a PepMap Neo Trap Cartridge (300 μm × 5 mm, 5 μm), while separation ran on an Easy-Spray™ PepMap™ Neo UHPLC analytical column (150 μm × 15 cm, 2 μm) held at 55 °C. The mobile phases consisted of A: 0.1% formic acid in water, and B: 0.1% formic acid in acetonitrile. Each injection carried 200 ng of peptide at a 2.5 μL/min flow; the effective separation gradient spanned 6.9 min within an 8 min total run. Detection was performed on an Orbitrap Astral high-resolution mass spectrometer operated in positive-ion DIA mode. Precursor scanning covered *m*/*z* 380–980, with MS1 resolution set to 240,000 at *m*/*z* 200; the DIA isolation window was 2 Th and HCD collision energy was 25%.

Raw MS files were analyzed in library-free mode with DIA-NN (v1.8.1). A deep-learning-predicted theoretical spectral library was built from the UniProt Rattus norvegicus reference proteome (UP000002494, 47,930 sequences), and the Match Between Runs (MBR) function was switched on to assemble an in-house library from the DIA datasets; this library was subsequently applied to reprocess every sample for protein identification and quantification. FDR at both the precursor-ion and protein levels was held strictly at 1% (<0.01). Quantification relied on the MaxLFQ algorithm embedded in DIA-NN, which derives protein intensities from log2 intensity ratios of Razor + Unique peptides; per-sample normalization was then achieved by dividing each intensity by the sample median. Sample reproducibility was gauged through principal-component analysis together with sample-correlation analysis. For proteins differing between the Model and High-dose cohorts, mean fold change (FC) across biological replicates was combined with Student’s *t*-test, with significance declared at FC ≥ 1.5 and *p* ≤ 0.05. Lastly, systematic annotation and hypergeometric-test enrichment—covering GO, KOG, KEGG pathways, protein domains and subcellular localization—were run on the differentially expressed proteins to clarify their biological roles and candidate regulatory mechanisms in a comprehensive manner [33,34].

### 4.11. Metabolomics Analysis of Rat Liver Tissue

In this project, untargeted metabolomics technology was employed. First, metabolites were extracted from 12 rat liver samples (High group and Model group, with 6 biological replicates per group). Thereafter, samples were analyzed by UPLC coupled to triple-quadrupole time-of-flight MS, acquiring data in both positive- and negative-ion modes. Separation ran on a Shimadzu LC-30A UHPLC fitted with a Waters ACQUITY Premier HSS T3 column (2.1 mm × 100 mm, 1.8 μm), held at 40 °C at a 0.4 mL/min flow with a 4 μL injection. The mobile phases were A: 0.1% formic acid in water and B: 0.1% formic acid in acetonitrile. The gradient program proceeded as: 0 min, 95% A/5% B; 2 min, 80% A/20% B; 5 min, 40% A/60% B; 6 min, 1% A/99% B; 7.5 min, 1% A/99% B; 7.6 min, 95% A/5% B; 10 min, 95% A/5% B.

MS data were acquired on a SCIEX TripleTOF 6600+ high-resolution instrument (Framingham, MA, USA)operated in both positive and negative electrospray ionization (ESI) modes. The ESI voltage was maintained at 5000 V (ESI+) and −4000 V (ESI−); ion source temperature: 550 °C (ESI+), 450 °C (ESI−); ion source gas 1 and gas 2: 50 psi and 60 psi; curtain gas: 35 psi. Declustering potential: +60 V (ESI+), −60 V (ESI−). MS1 scan range *m*/*z* 50–1000 Da, MS2 scan range *m*/*z* 25–1000 Da; collision energy 30 V with collision energy spread 15 V.

Raw files were first converted into mzML format through ProteoWizard, after which XCMS software 4.6.3 was applied for peak detection, sample-to-sample alignment and retention-time recalibration. Data preprocessing was completed through KNN imputation and SVR normalization. After obtaining quantitative metabolite data, sample relationships were assessed and models were constructed using principal component analysis and orthogonal partial least squares discriminant analysis. Differential metabolites were screened using the criteria of VIP > 1, *p* < 0.05, and |log2FC| > 1. Systematic bioinformatics analysis was then conducted on the differential metabolites, including KEGG pathway annotation and enrichment analysis, HMDB functional annotation and enrichment analysis, MSEA metabolite set enrichment analysis, and disease association analysis of differential metabolites, to comprehensively elucidate the biological pathways and potential functional mechanisms involving the differential metabolites.

### 4.12. Identification of Components Using UPLC-Q/TOF-MS/MS

#### 4.12.1. Preparation of Extract

The PAFFB fermentation broth was lyophilized to a dry powder. A precisely weighed 20 mg aliquot of this powder was taken up in 5 mL of 50% methanol. After centrifugation at 12,000 r/min for 10 min, the supernatant was passed through a 0.22 μm microporous filter, and the resulting filtrate was subjected to UPLC-Q-TOF-MS/MS for constituent identification and structural characterization.

#### 4.12.2. Plasma Specimen Preparation and Workup

Prior to the formal trial, animals underwent a 12 h fast with drinking water available ad libitum; throughout the dosing period, water remained accessible while food was withdrawn. Twelve rats were allocated at random to either a control cohort receiving normal saline or a treatment cohort receiving PAFFB. The lyophilized PAFFB powder was delivered by oral gavage at 3 g·kg^−1^. Orbital-vein blood was drawn at 15 min, 0.5, 1, 2, 4, 6, 8 and 12 h post-dose. Within each cohort, 300 µL of pooled plasma was combined with 1.5 mL of 50% methanol for protein precipitation, then spun at 12,000 rpm for 15 min at 4 °C. The supernatant was dried under a nitrogen stream, and the residue was redissolved in 200 µL of 50% methanol after 2 min of vortex mixing. After centrifugation, the supernatant was collected for analysis. Blank plasma samples were subjected to the same procedure [35,36].

#### 4.12.3. UPLC/Q-TOF-MS/MS Analytical Conditions

Liquid Chromatography Conditions [37]: Chromatographic separation was carried out on a Waters ACQUITY UPLC HSS T3 column (2.1 × 100 mm, 1.7 μm) held at 40 °C. A 3 μL aliquot was injected, and the flow rate was maintained at 0.3 mL/min. The mobile phase was composed of solvent A (0.1% formic acid in water) and solvent B (acetonitrile), and gradient elution proceeded as follows: 5–50% B from 0.01 to 35 min, 50–95% B from 35 to 40 min, and 95% B from 40 to 45 min.

MS Conditions [38]: Analyses were carried out on a TripleTOF™ 5600 mass spectrometer (AB Sciex, Framingham, MA, USA) fitted with an electrospray ionization (ESI) source. Data were acquired in TOF MS and Product Ion modes, each with a 0.3 s scan time, over a mass range of 50–1250 *m*/*z*. The ion source was operated with the following settings: GS1 (nebulizer gas) 50 mL/min, GS2 (desolvation gas) 50 mL/min, CUR (curtain gas) 30 mL/min, and TEM (desolvation temperature) 550 °C. The ISVF (ion spray voltage) was 5500 V in positive ion mode and −4500 V in negative ion mode, while the DP (declustering potential) was ±100 V. Collision energy was 10 eV for TOF MS and 30 eV for Product Ion scans, with a CES (collision energy spread) of ±15 V. The IRD (ion release delay) was 67 and the IRW (ion beam width) was 25.

### 4.13. Cell Culture and In Vitro Pharmacological Assays

Hepa1-6 cells were purchased from Suzhou Haixing Biotechnology Co., Ltd. (Suzhou, China) and maintained in DMEM supplemented with 10% FBS at 37 °C under 5% CO_2_. Phytolaccagenin (purity ≥ 98%) was first dissolved in DMSO and then diluted in culture medium to the desired working concentrations (50–150 μM; final DMSO ≤ 0.1%). For the CCK-8 assay, cells were plated in 96-well plates and exposed to treatment for 12, 24, or 48 h. Colony formation ability was evaluated by crystal violet staining following 10–14 days of culture. Cell migration was examined by a scratch wound assay, with images recorded at 0 and 24 h. Apoptosis was assessed by Annexin V-FITC/PI double staining and flow cytometry. Intracellular ROS levels were determined with the DCFH-DA probe using flow cytometry and fluorescence microscopy in parallel. Lipid peroxidation was visualized with the C11-BODIPY581/591 probe. All experiments were conducted in triplicate.

CCK-8 assay: Hepa1-6 cells were plated into 96-well plates at 1 × 10^4^ cells per well and incubated overnight to allow attachment. Following exposure to phytolaccagenin (50, 100, or 150 μM) for 12, 24, or 48 h, each well received 10 μL of CCK-8 reagent, and the plates were kept at 37 °C for 1 h protected from light. Optical density was recorded at 450 nm on a microplate reader (Feyond-A300, Hangzhou City, Zhejiang, China). The percentage of viable cells was computed as (A_treated_ − A_blank_)/(A_control_ − A_blank_) × 100%. Three independent biological replicates, each with six technical replicates, were conducted for this assay.

Colony formation assay: Cells were plated into 6-well plates at 500 cells per well and maintained for 24 h prior to exposure to phytolaccagenin (0, 50, 100, and 150 μM) over a 14-day period, during which the medium was replaced every 2 days. Following fixation with 4% paraformaldehyde for 15 min, colonies were stained with 0.1% (w/v) crystal violet for 20 min and then rinsed with PBS. Images were captured under a microscope, and the colony area was quantified with ImageJ software 1.54f.

Migration assay: Cells were plated into 6-well plates and grown until 90% confluence was reached. A linear scratch was made using a 200 μL pipette tip, after which detached cells were eliminated by two PBS washes. The cells were then maintained in serum-free medium supplemented with phytolaccagenin (0–150 μM). Using an inverted microscope (BZ-X810, Keyence, Osaka, Japan), images of identical wound regions were recorded at 0 and 24 h, and the wound area was measured with ImageJ. The migration rate (%) was computed according to the formula [(wound area at 0 h–wound area at 24 h)/wound area at 0 h] × 100%.

Apoptosis assay: Cells were plated into 6-well plates, exposed to phytolaccagenin (0–150 μM) for 24 h, then collected and rinsed with PBS. Apoptotic cells were identified using an Annexin V-FITC/PI double staining kit (APExBIO, Houston, TX, USA, Cat No: K2003) in accordance with the supplier’s protocol, and the samples were examined on a flow cytometer (Thermo Fisher Attune NxT, Waltham, MA, USA) within 1 h, with a minimum of 10,000 events acquired per sample. The percentages of early (Annexin V^+^/PI^−^) and late (Annexin V^+^/PI^+^) apoptotic cells were summed as the total apoptotic rate.

ROS detection: After treatment, cells were incubated with 10 μM DCFH-DA probe (Jiangsu Keygen Biotech Corp., Ltd., Nanjing, China, Cat No: KGA7308-100) at 37 °C for 30 min in the dark, washed twice with serum-free medium, and analyzed by flow cytometry (Thermo Fisher Attune NxT, USA) for mean fluorescence intensity. Fluorescence images were also acquired with a fluorescence microscope (BZ-X810, Keyence, Osaka, Japan) for visual confirmation.

Lipid peroxidation assay: Treated cells were stained with 1 μM C11-BODIPY581/591 probe (Beyotime, Shanghai, China, Cat No: S0043S) at 37 °C for 30 min in the dark, washed, and analyzed by fluorescence microscope (BZ-X810, Keyence, Osaka, Japan).

All in vitro experiments were performed in at least three independent biological replicates.

### 4.14. In Vivo Anti-Tumor Assay in C57BL/6 Mice

Male C57BL/6 mice aged 6–8 weeks, weighing 18–22 g, were selected as experimental animals. Prior to the formal experiment, all mice were acclimatized for 3 days with free access to food and water to ensure adequate environmental adaptation. For tumor inoculation, mice in the experimental groups (except the control group) were subcutaneously injected in the right scapular region with 100 μL of a cell suspension containing Hepa1-6 cells at a concentration of 6 × 10^7^ cells/mL, which was prepared by suspending the cells in 5% DMEM and mixing with Matrigel at a 1:1 ratio. Tumor-bearing mice were then randomly divided into five groups (*n* = 6 per group): model group, positive group, Phytolaccagenin L (5 mg/kg), Phytolaccagenin-M (10 mg/kg), and Phytolaccagenin-H (20 mg/kg). An additional six untreated mice served as the control group. For drug intervention, the Phytolaccagenin-treated groups received daily intraperitoneal injections of the corresponding doses, while the positive control group received cyclophosphamide (25 mg/kg) intraperitoneally every two days. The blank control and model groups were given an equal volume of normal saline daily. Treatment was initiated when the mean tumor volume reached approximately 100 mm^3^ (approximately 7 days after tumor-cell inoculation). Tumor volume was measured every 2 days with a digital caliper and calculated as V = (length × width^2^)/2. The mice received the indicated treatments for 14 consecutive days; at the end of the treatment period (day 14 after treatment initiation), the mice were sacrificed by CO_2_ euthanasia, and the following parameters were assessed: (i) body weight changes; (ii) tumor volume; (iii) peripheral blood routine parameters; (iv) tumor weight and gross tumor images; and (v) histopathological examination of tumor tissues as well as heart, liver, spleen, lung, and kidney by H&E staining. All experimental procedures were conducted in accordance with institutional animal care guidelines.

### 4.15. qRT-PCR

Total RNA was isolated from the right median hepatic lobe of rats (obtained at the scheduled sacrifice from the same animal batch) with the Animal Total RNA Isolation Kit (Cat. No. RE-03011; Foregene Biotechnology Co., Ltd., Chengdu, China), following the supplier’s protocol. First-strand cDNA was then generated with the Evo M-MLV RT Kit with gDNA Clean for qPCR (Cat. No. AG11728; Accurate Biology (Hunan) Co., Ltd., Changsha, China). Quantitative PCR was carried out using the SYBR^®^ Green Premix Pro Taq HS qPCR Kit (Cat. No. AG11701; Accurate Biology (Hunan) Co., Ltd., Changsha, China). The relative abundance of target transcripts was computed based on the 2^−ΔΔCt^ approach, with data calibrated against an internal reference gene. Each sample was run in triplicate, with 3 biological replicates per group. The primer sequences used for qRT-PCR were as follows: *Cd3d*, GTCCGGTGACTTGCCTCTAC(F), CTAGATGCCTGATGCTGGTGT(R); *Akr1c1*, TTCGTTATCAGCTGGAGCGT(F), TCTAGAACCACTCATGTAACGGA(R); *Hexd*, ACGACGTGGGAAGAAGTCACA(F), CTCTGTAACTTCCGAGGGGC(R); *Cyp7a1*, GGGCAGGCTTGGGAATTTTG(F), CTAATCCAGTCGCTAGGGCG(R); *Cd19*, ATGCCATCTCCTCTCCCTGT(F), AATCTCCTAGCAGGGTCGGT(R); *Cd22*, CTCGAACACAGGGGGAAACA(F), CAGAGGCCACCGCGTATTTA(R); *Il-6*, AGAGACTTCCAGCCAGTTGC(F), AGTCTCCTCTCCGGACTTGT(R); *Tnf-α*, GGCTTTCGGAACTCACTGGA(F), GGGAACAGTCTGGGAAGCTC(R); *Il-1b*, TCCTCTGTGACTCGTGGGAT(F), TCAGACAGCACGAGGCATTT(R); *β-actin*, GACATGCCGCCTGGAGAAAC(F), AGCCCAGGATGCCCTTTAGT(R).

### 4.16. Western Blotting

Rat liver tissues were homogenized on ice in RIPA lysis buffer supplemented with 1% PMSF and phosphatase inhibitors, and protein concentrations were quantified with a BCA protein assay kit. Lysates were adjusted to a uniform concentration using RIPA buffer and 5× loading buffer, followed by denaturation at 100 °C for 15 min. Equal protein amounts (20 μg per lane) were resolved by SDS-PAGE on 10% polyacrylamide gels and then electrotransferred to PVDF membranes. Membranes were blocked for 2 h at room temperature in TBST containing 5% skim milk, rinsed five times with 1× TBST (10 min each), and incubated overnight at 4 °C with primary antibodies (1:1000) against TLR-4, MyD88, p65, p-p65, p38, p-p38, CYP7A1, CYP4B1, and β-Actin. After five additional 10 min washes in 1× TBST, the membranes were probed with HRP-conjugated goat anti-rabbit IgG secondary antibody for 90 min at room temperature. Protein bands were visualized using ECL substrate, and relative protein expression was quantified with a chemiluminescence imaging system (Image Lab™, Bio-Rad, Hercules, CA, USA), with band intensities normalized to β-Actin. Three independent replicates were performed for all experiments.

### 4.17. Data and Statistical Analysis

Statistical analyses were carried out with SPSS 26 (SPSS Science Inc., Chicago, IL, USA) and Prism 8.0 (GraphPad Software, La Jolla, CA, USA). Measurement data are summarized as the mean ± SD. Comparisons involving only two groups—namely, the differential proteomic analysis and the comparison of individual metabolites between the Model and High-dose groups—were performed using the independent-samples *t*-test. For comparisons among more than two groups, one-way analysis of variance (ANOVA) followed by Tukey’s HSD post-hoc test was employed. Statistical significance was defined as *p* < 0.05, *p* < 0.01, or *p* < 0.001.

## 5. Conclusions

In conclusion, this study establishes PAFFB as a multi-target anti-HCC agent that systematically attenuates DEN-induced metabolic reprogramming and inflammatory dysregulation via coordinated regulation of the PPARα/FXR/CYP7A1 axis and the TLR-4/Myd88/NF-κB/MAPK signaling cascade. Importantly, by integrating UPLC-Q-TOF-MS/MS chemical profiling with subsequent bioactivity validation, we successfully pinpointed Phytolaccagenin as one of the bona fide active principles among the 21 blood-absorbed components. The convergence of in vitro mechanistic data (ROS-driven lipid peroxidation, apoptosis induction, and migration suppression) and in vivo efficacy data (syngeneic tumor growth inhibition with favorable safety) provides compelling evidence that Phytolaccagenin directly contributes to the observed anti-HCC effects of PAFFB. Collectively, our findings not only support the continued development of PAFFB as a holistic adjuvant therapy, but also offer a lead compound for future drug discovery efforts. This work underscores the value of systems pharmacology-combining multi-omics, chemical profiling, and targeted validation-as a powerful strategy for elucidating and translating the therapeutic potential of complex traditional Chinese medicine preparations.

## Figures and Tables

**Table 1:** Identification of 120 chemical components of PAFFB.

No.	t_R_ (min)	*m*/*z*		Mass Error (ppm)	Fragment Ions	Molecular Formula	Tentative Identification
1	0.91	203.2230	[M+H]^+^	−0.2	112.1143, 84.0823	C_10_H_26_N_4_	Spermine
2	0.94	146.1648	[M+H]^+^	−2.6	110.0070, 84.0824, 68.9851	C_7_H_19_N3	Spermidine
3	0.97	112.0870	[M+H]^+^	1.1	95.0607, 81.0448, 68.0505	C_5_H_9_N_3_	Histamine
4	1.01	188.1749	[M+H]^+^	−2.4	114.0917, 84.0821, 72.0825	C_9_H_21_N_3_O	N8-Acetylspermidine
5	1.02	147.1124	[M+H]^+^	−3.1	111.0067, 84.0814, 69.9831	C_6_H_14_N_2_O_2_	L-Lysine monohydrochloride
6	1.2	189.1596	[M+H]^+^	−0.9	84.0812, 60.0814, 56.0514	C_9_H_20_N_2_O_2_	N6,N6,N6—Trimethyl-L-lysine
7	1.38	136.0619	[M+H]^+^	1.2	119.0360, 92.0261, 65.0156	C_5_H_5_N_5_	Adenine
8 *	1.39	138.0551	[M+H]^+^	0.8	94.0647, 79.0422, 66.0351	C_7_H_7_NO_2_	Trigonelline
9	1.40	116.0707	[M+H]^+^	0.9	70.0671	C_5_H_9_NO_2_	Proline
10	1.44	130.0862	[M+H]^+^	−0.3	84.0819, 68.0505, 56.0516	C_6_H_11_NO_2_	D-(+)-Pipecolinic acid
11	1.44	118.0864	[M+H]^+^	1.0	72.0836, 55.0573	C_5_H_11_NO_2_	L-Valine
12	1.51	150.0583	[M+H]^+^	0	61.0129, 56.0524	C_5_H_11_NO_2_S	L-(−)-Methionine
13	1.54	182.0812	[M+H]^+^	0.3	136.0791, 107.0509, 95.0522, 91.0568, 77.0407	C_9_H_11_NO_3_	L-Tyrosine
14	1.54	180.1019	[M+H]^+^	−0.3	-	C_10_H_13_NO_2_	Salsolinol
15	1.56	130.0499	[M+H]^+^	−0.1	-	C_5_H_7_NO_3_	L-Pyroglutamic acid
16	1.65	138.0912	[M+H]^+^	−0.9	121.0671, 103.0568, 91.0568, 77.0413	C_8_H_11_NO	Tyramine
17	1.73	117.0201	[M−H]^−^	6.8	99.9228, 73.0318	C_4_H_6_O_4_	Succinic acid
18	1.76	132.1020	[M+H]^+^	0.4	86.0986, 69.0723, 57.0603	C_6_H_13_NO_2_	DL-Norleucine
19	2.49	180.1020	[M+H]^+^	0.6	127.0522, 115.0531, 91.0540, 77.0380	C_10_H_13_NO_2_	Salsolinol
20	2.50	199.1077	[M+H]^+^	−0.1	153.1072, 123.0926, 70.0663	C_9_H_14_N_2_O_3_	3-(1-hydroxyethyl)-2,3,6,7,8,8a-hexahydropyrrolo [1,2-a]pyrazine-1,4-dione
21	2.75	144.0476	[M+H]^+^	−0.9	-	C_6_H_9_NOS	4-Methyl-5-thiazole ethanol
22	2.86	166.0864	[M+H]^+^	0.9	120.0812, 103.0553, 77.0406	C_9_H_11_NO_2_	phenylalanine
23 *	2.86	120.0808	[M+H]^+^	0.3	91.0554, 77.0401	C_8_H_9_N	3-ethenylaniline
24	3.21	197.0457	[M−H]^−^	1	135.0406, 123.0445, 72.9941	C_9_H_10_O_5_	Syringic acid
25	3.30	341.0845	[M−H]^−^	−9.6	341.0795, 253.0993, 180.0762, 148.0395	C_15_H_18_O_9_	Caffeic acid 3-o-β-d-glucuronide
26	3.45	153.0192	[M−H]^−^	−0.6	109.0305, 108.0224, 91.0157	C_7_H_6_O_4_	3,4-dihydroxybenzoic acid
27	3.69	315.0707	[M−H]^−^	−4.7	193.0609, 131.0317, 89.0261	C_13_H_16_O_9_	Protocatechuic acid 3-O-β-D-glucoside
28	3.75	153.0560	[M−H]^−^	1.9	123.0452, 108.0250, 93.0358	C_8_H_10_O_3_	2-(3,4-Dihydroxyphenyl)ethanol
29	4.38	217.0512	[M+H]^+^	7.4	146.0607	C_12_H_8_O_4_	Majudin
30	4.40	135.0457	[M−H]^−^	3.9	117.0257, 77.0389	C_8_H_8_O_2_	4′-hydroxyacetophenone
31 *	4.41	163.0406	[M−H]^−^	3.0	132.0274, 93.0352	C_9_H_8_O_3_	Cis-4-coumaric acid
32	4.77	222.0773	[M−H]^−^	0.7	180.0744, 163.0384, 119.0496	C_11_H_13_NO_4_	N-Acetyl-L-tyrosine
33	4.95	137.0252	[M−H]^−^	6.0	93.0353, 75.0247, 65.0424	C_7_H_6_O_3_	3,4-dihydroxybenzaldehyde
34	4.99	339.0725	[M−H]^−^	1.0	221.0441, 189.0547, 161.0228, 149.0585	C_15_H_16_O_9_	Esculin
35	5.00	139.0389	[M+H]^+^	−0.4	93.0337, 77.0408, 65.0412	C_7_H_6_O_3_	4-Hydroxybenzoic acid
36	6.07	179.0351	[M−H]^−^	0.8	137.0225, 107.0082	C_9_H_8_O_4_	Caffeic acid
37	6.25	177.0197	[M−H]^−^	2	149.0134, 133.0281, 105.0300, 89.0419	C_9_H_6_O_4_	Esculetin
38	6.36	167.0353	[M−H]^−^	2.1	152.0107, 108.0223, 91.0200	C_8_H_8_O_4_	3-hydroxy-4-methoxybenzoic acid
39 *	6.75	229.0989	[M−H]^−^	2.7	156.0786, 142.0658, 116.0506	C_13_H_14_N_2_O_2_	1H-Pyrido [3,4-b]indole-3-carboxylicacid, 2,3,4,9-tetrahydro-1-methyl
40	8.32	487.1478	[M−H]^−^	4.2	235.0442, 193.0484, 178.0257, 149.0591, 134.0362	C_21_H_28_O_13_	(2E)-3-[4-({2-O-[(2S,3R,4R)-3,4-Dihydroxy-4-(hydroxymethyl)tetrahydro-2-furanyl]-beta-D-glucopyranosyl}oxy)-3-methoxyphenyl]acrylic acid
41 *	8.94	343.0820	[M−H]^−^	−0.8	255.1029, 226.0619, 109.0278	C_18_H_16_O_7_	Xanthomicrol
42	9.13	383.0989	[M−H]^−^	1.4	263.0636, 220.0428, 203.0361, 163.0372	C_17_H_20_O_10_	Calycanthoside
43	9.31	635.1632	[M+Na]^+^	7.8	617.1463, 515.1051, 467.1156, 353.0869, 191.0307	C_27_H_32_O_16_	β-D-Glucopyranoside, 4-carboxy-2-methoxyphenyl 2-O-[(2S,3R,4S)-tetrahydro-3,4-dihydroxy-4-[[(4-hydroxy-3-methoxybenzoyl)oxy]methyl]-2-furanyl]
44 *	9.46	147.0463	[M−H]^−^	7.7	117.0327, 101.0393, 77.0419	C_9_H_8_O_2_	Trans-cinnamic acid
45 *	9.46	165.0568	[M−H]^−^	6.5	147.0486, 117.0345, 101.0409	C_9_H_10_O_3_	3-(3,4-dihydroxyphenyl)propanoic acid
46 *	9.46	353.1018	[M−H]^−^	−3.5	165.0552, 147.0449, 119.0506, 103.0568	C_20_H_18_O_6_	Erythrinin G
47 *	10.04	195.0671	[M−H]^−^	4.1	135.0446, 121.0298, 108.0226	C_10_H_12_O_4_	Rhizonic acid
48	10.55	206.0828	[M−H]^−^	2.4	164.0674, 147.0427, 103.0558, 91.0557	C_11_H_13_NO_3_	N-Acetyl-L-phenylalanine
49 *	10.84	319.0470	[M−H]^−^	3.4	239.0898, 193.0836, 171.1026, 125.0971	C_15_H_12_O_8_	Ampelopsin
50	11.09	228.0632	[M+Na]^+^	0.5	168.0779	C_11_H_11_NO_3_	Indolelactic acid
51	11.11	309.1813	[M+H]^+^	1.2	223.1424, 177.1374, 121.0747	C_16_H_24_N_2_O_4_	Ubenimex
52	11.16	204.0671	[M−H]^−^	2.6	186.0535, 158.0595, 142.0662, 130.0656, 116.0507	C_11_H_11_NO_3_	dl-3-Indolelactic acid
53	11.35	771.2036	[M−H]^−^	6.1	625.1352, 591.1555, 301.0368, 271.0250, 178.9993	C_33_H_40_O_21_	Quercetin 3-O-robinoside-7-O-glucoside
54	11.63	757.1867	[M−H]^−^	4.6	577.1261, 300.0259, 271.0206	C_32_H_38_O_21_	3-[(2S,3R,4S,5R,6R)-4-[(2S,3R,4S,5S,6R)-4,5-dihydroxy-6-(hydroxymethyl)-3-[(2S,3R,4S,5R)-3,4,5-trihydroxyoxan-2-yl]oxyoxan-2-yl]oxy-3,5-dihydroxy-6-(hydroxymethyl)oxan-2-yl]oxy-2-(3,4-dihydroxyphenyl)-5,7-dihydroxychromen-4-one
55	11.64	465.1062	[M+H]^+^	7.4	303.0520, 257.0439, 229.0529, 153.0193, 137.0256	C_21_H_20_O_12_	Quercetin-3-O-glucoside
56	11.78	595.1331	[M−H]^−^	4.5	300.0280, 271.0252, 255.0300	C_26_H_28_O_16_	2-(3,4-dihydroxyphenyl)-5,7-dihydroxy-3-[(2S,3R,4S,5S,6R)-3,4,5-trihydroxy-6-[[(2S,3R,4S,5R)-3,4,5-trihydroxyoxan-2-yl]oxymethyl]oxan-2-yl]oxychromen-4-one
57	12.00	245.0935	[M−H]^−^	1.2	159.0932, 142.0677, 116.0512, 98.0286	C_13_H_14_N_2_O_3_	N-acetyltryptophan
58	12.00	357.0985	[M−H]^−^	1.4	251.0690, 145.0262, 91.0231	C_19_H_18_O_7_	3-[2-(2-hydroxy-4-methoxyphenyl)-2-oxoethyl]-6,7-dimethoxy-3-hydroisobenzofuran-1-one
59	12.21	313.1091	[M+H]^+^	6.4	277.0804, 249.0933, 231.0821, 203.0858	C_18_H_16_O_5_	5,7,8-Trimethoxyflavone
60	12.35	351.0866	[M−H]^−^	−2.3	189.0543, 159.0473, 137.0238	C_20_H_16_O_6_	Coryaurone A
61	12.54	779.2070	[M+Na]^+^	8.3	633.1410, 493.1553, 347.0959, 313.0923, 203.0537	C_33_H_40_O_20_	5,7-dihydroxy-2-[4-[(2S,3R,4S,5S,6R)-3,4,5-trihydroxy-6-(hydroxymethyl)oxan-2-yl]oxyphenyl]-3-[(2S,3R,4S,5S,6R)-3,4,5-trihydroxy-6-[[(2R,3R,4R,5R,6S)-3,4,5-trihydroxy-6-methyloxan-2-yl]oxymethyl]oxan-2-yl]oxychromen-4-one
62	12.45	637.1804	[M−H]^−^	4.7	413.1103, 301.1022, 223.0597, 137.0246	C_29_H_34_O_16_	Ombuoside
63	12.62	463.0891	[M−H]^−^	1.8	300.0272, 271.0241, 255.0278	C_21_H_20_O_12_	quercetin 3-O-β-D-glucofuranoside
64	13.15	603.1369	[M+Na]^+^	8	471.1032, 317.0900, 299.0819, 215.0553, 185.0442	C_26_H_28_O_15_	Leucoside
65	13.63	165.0911	[M+H]^+^	0.7	55.0556	C_10_H_12_O_2_	Isoeugenol
66 *	13.90	391.1402	[M−H]^−^	1	343.1212, 311.0950, 195.0665, 165.0538	C_20_H_24_O_8_	Dihydroresveratrol 3-O-glucoside
67	14.48	447.0943	[M−H]^−^	2.1	285.0402, 255.0263, 227.0328	C_21_H_20_O_11_	Astragalin
68	14.5	601.1574	[M+Na]^+^	7.6	437.1102, 349.0942, 275.0450	C_27_H_30_O_14_	Genkwanin 5-O-beta-primeveroside
69	14.6	315.1246	[M−H]^−^	2.4	300.1053, 255.0673, 209.0583, 121.0302	C_18_H_20_O_5_	[1-hydroxy-1-(4-methoxyphenyl)propan-2-yl] 4-methoxybenzoate
70	14.93	187.0982	[M−H]^−^	3.1	123.0791, 95.0577, 61.9907	C_9_H_16_O_4_	azelaic acid
71	15.93	515.1207	[M−H]^−^	2.4	353.0887, 191.0565, 179.0331, 173.0457	C_25_H_24_O_12_	Isochlorogenic acid A
72	16.85	589.2708	[M−H]^−^	9.0	412.2129, 331.1857, 161.0423, 119.0340	C_31_H_42_O_11_	Pre-schisanartanin B
73	17.73	314.1402	[M+H]^+^	4.9	177.0541, 145.0284, 121.0651, 103.0559	C_18_H_19_NO_4_	Moupinamide
74	17.73	336.1234	[M]	−0.6	320.0997, 278.0837	C_20_H_18_NO_4_^+^	berberine
75	18.56	273.0759	[M+H]^+^	0.5	185.1072, 168.0834, 143.0737	C_15_H_12_O_5_	Naringenin chalcone
76	18.63	811.4156	[M−H]^−^	4.3	649.3628, 499.3064	C_41_H_64_O_16_	esculentoside F
77	18.72	303.0523	[M+H]^+^	7.8	303.0503, 257.0456, 229.0503, 153.0173	C_15_H_10_O_7_	Viscidulin I
78	18.96	301.0360	[M−H]^−^	2.1	245.0448, 227.0328, 178.9978, 151.0032, 121.0296	C_15_H_10_O_7_	Quercetin
79	19.39	1149.5397	[M−H]^−^	4.8	1103.5451, 957.4832, 941.4836, 795.4329	C_54_H_86_O_26_	esculentoside N
80	20.34	317.0683	[M+H]^+^	8.5	302.0441, 274.0489, 229.0507, 161.0614	C_16_H_12_O_7_	2-(3,4-dihydroxyphenyl)-5,7-dihydroxy-3-methoxychromen-4-one
81	21.01	1133.5426	[M−H]^−^	3.6	925.4912, 779.4289, 617.3684, 599.3605	C_54_H_86_O_25_	phytolaccasaponin N-5
82	21.02	285.0774	[M−H]^−^	1.9	252.0405, 223.0393, 149.9970, 123.0082	C_16_H_14_O_5_	(2R,3R)-3,7-dihydroxy-6-methoxy-2-phenyl-2,3-dihydrochromen-4-one
83	21.05	345.0620	[M−H]^−^	1.0	330.0333, 315.0165, 287.0147	C_17_H_14_O_8_	viscidulin III
84 *	22.07	287.0564	[M+H]^+^	4.7	229.0498, 153.0179, 121.0284	C_15_H_10_O_6_	kaempferol
85	22.32	285.0407	[M−H]^−^	0.9	285.0410, 255.0274, 211.0382	C_15_H_10_O_6_	aureusidin
86	22.33	985.4682	[M−H]^−^	3.2	939.4696, 793.4073, 671.2644, 531.2966	C_48_H_74_O_21_	esculentoside G
87	22.82	679.3737	[M−H]^−^	5.6	635.3843, 517.3191, 473.3275, 425.3088	C_36_H_56_O_12_	Phytolaside A
88	22.97	255.0663	[M−H]^−^	−0.1	213.0560, 185.0620, 141.0700, 123.0158	C_15_H_12_O_4_	5,7-Dihydroxyflavanone
89 *	23.42	649.3627	[M−H]^−^	5.2	605.3735, 517.3156, 473.3268	C_35_H_54_O_11_	esculentoside E
90	23.75	663.3783	[M−H]^−^	4.9	543.3385, 501.3259, 457.3352	C_36_H_56_O_11_	Esculentoside B
91	24.19	633.3657	[M−H]^−^	2.0	633.3674, 589.3765	C_35_H_54_O_10_	esculentoside O
92	24.58	329.0669	[M−H]^−^	0.5	299.0205, 271.0249, 243.0296, 199.0430	C_17_H_14_O_7_	Ombuin
93	24.86	361.0947	[M+H]^+^	8	345.0658, 303.0512, 269.0469, 257.0454, 229.0523, 121.0301	C_18_H_16_O_8_	5,7-dihydroxy-2-(3-hydroxy-4-methoxyphenyl)-3,6-dimethoxychromen-4-one
94 *	24.97	989.5040	[M+H]^+^	8.9	533.3642, 497.3278, 241.0692	C_48_H_76_O_21_	Esculentoside H
95	25.09	359.0777	[M−H]^−^	1.1	344.0654, 329.0326, 314.0057, 286.0116, 258.0164, 202.0260	C_18_H_16_O_8_	jaceidin
96	25.14	1017.496	[M−H]^−^	5.4	971.4984, 825.4395, 663.3842, 531.3333	C_49_H_78_O_22_	Esculentoside I
97 *	25.15	825.4377	[M−H]^−^	6.9	487.3451, 337.0803, 265.9478	C_42_H_66_O_16_	Esculentoside A
98 *	26.30	501.3252	[M+H]^+^	8.3	465.3027, 437.3060, 391.3012, 311.2050, 231.1388	C_30_H_44_O_6_	3,19-Dihydroxy-6,23-dioxo-12-ursen-28-oic acid
99 *	26.83	517.3175	[M−H]^−^	0.7	473.3282	C_30_H_46_O_7_	Jaligonic acid
100	26.90	299.0934	[M+H]^+^	6.8	283.0629, 268.0385, 241.0538, 213.0582, 151.0388	C_17_H_14_O_5_	5-hydroxy-6,7-dimethoxyflavone
101	28.11	971.4904	[M−H]^−^	4.8	839.4557, 677.3964, 659.3832, 545.3500	C_48_H_76_O_20_	Esculentoside L
102 *	28.31	809.4358	[M−H]^−^	3.6	763.4492, 471.3488, 337.0775	C_42_H_66_O_15_	Esculentoside C
103	28.54	501.3240	[M−H]^−^	3.7	457.3353, 379.3029	C_30_H_46_O_6_	3,4-Secocucurbita-4,24-diene-3,26,29-trioic acid
104	28.70	297.0781	[M+H]^+^	7.7	282.0533, 225.0555, 180.0079, 139.0554	C_17_H_12_O_5_	(7-hydroxy-4-oxo-2-phenylchromen-5-yl) acetate
105	29.33	255.0664	[M−H]^−^	0.3	169.0620, 145.0656, 65.0056	C_15_H_12_O_4_	2,2′,4′-Trihydroxychalcone
106	29.36	285.0776	[M+H]^+^	6.4	285.0808, 270.0535, 224.0501	C_16_H_12_O_5_	Calycosin
107	29.85	693.3895	[M−H]^−^	5.8	647.3850, 515.3240, 286.1151	C_37_H_58_O_12_	Esculentoside D
108	29.89	269.0459	[M−H]^−^	1.3	223.0355, 169.0646, 116.9255	C_15_H_10_O_5_	Genistein
109	30.67	329.1041	[M+H]^+^	6.5	281.0496, 239.0765, 195.0293, 165.0717	C_18_H_16_O_6_	Isoamericanin A
110	30.35	821.4015	[M−H]^−^	6.1	483.3127, 377.2490, 337.0783, 277.9076	C_42_H_62_O_16_	Glycyrrhizic acid
111 *	31.06	531.3347	[M−H]^−^	3.6	485.3306, 453.3039, 427.2902, 377.2524	C_31_H_48_O_7_	Phytolaccagenin
112 *	34.05	515.3396	[M−H]^−^	3.4	437.3106, 411.2929, 389.2794, 361.2464, 279.1911	C_31_H_48_O_6_	phytolaccagenic acid
113	36.59	487.3447	[M−H]^−^	3.6	146.9672, 129.9739	C_30_H_48_O_5_	Arjunic acid
114 *	37.47	485.3288	[M−H]^−^	3.1	441.3376, 393.3245, 129.9746	C_30_H_46_O_5_	Actinidic acid
115	38.16	431.2040	[M−H]^−^	−8.2	363.2112, 295.2272, 185.1171	C_24_H_32_O_7_	Gomisin A
116	39.48	483.3135	[M−H]^−^	3.9	437.3120, 407.2906	C_30_H_44_O_5_	Fupenzic acid
117	40.87	277.2175	[M−H]^−^	0.6	/	C_18_H_30_O_2_	2-Monolinolein
118	41.00	227.2019	[M−H]^−^	0.9	/	C_14_H_28_O_2_	Myristic acid
119	41.3	353.2693	[M−H]^−^	−1.2	279.2464, 73.0291	C_21_H_38_O_4_	1-Monolinolein
120 *	41.67	371.1534	[M−H]^−^	9.1	339.1664, 253.2143, 223.0247	C_21_H_24_O_6_	Arctigenin

Note: “*” represents the components absorbed into the blood.

## Data Availability

The original contributions presented in this study are included in the article. Further inquiries can be directed to the corresponding author.

## Abbreviations

HCC, hepatocellular carcinoma; PAFFB, *Phytolacca acinosa* fruit fermentation broth; DEN, diethylnitrosamine; UPLC-Q-TOF-MS/MS, ultra-high performance liquid chromatography–mass spectrometry; SD, Sprague Dawley; SPF, specific pathogen-free; CTX, cyclophosphamide; SP, streptavidin-peroxidase; PPARα, peroxisome proliferator-activated receptor alpha; FXR, farnesoid X receptor; CYP7A1, cholesterol 7α-hydroxylase; GO, Gene Ontology; DEGs, differentially expressed genes; BP, Biological Process; CC, Cellular Component; MF, Molecular Function.
